# Impact of Oxygen Release from Bentonite on Microbial Activity, Mineralogy, and Steel Corrosion

**DOI:** 10.1021/acs.est.5c08788

**Published:** 2025-11-19

**Authors:** Natalia Jakus, Pranav Vivek Kulkarni, Carolin L. Dreher, Sylvie Bruggmann, Daniel Grolimund, Andreas Kappler, Nikitas Diomidis, Stefano Mischler, Rizlan Bernier-Latmani

**Affiliations:** † Environmental Microbiology Laboratory, École Polytechnique Fédérale de Lausanne (EPFL), Station 6, CH-1015 Lausanne, Switzerland; ‡ Tribology and Interfacial Chemistry Group, École Polytechnique Fédérale de Lausanne (EPFL), Station 12, CH-1015 Lausanne, Switzerland; § Geomicrobiology Group, 9188 University of Tübingen, Schnarrenbergstrasse 94-96, D-72074 Tübingen, Germany; ∥ Institute of Earth Sciences, University of Lausanne, Quartier UNIL-Mouline, Bâtiment Géopolis, CH-1015 Lausanne, Switzerland; ⊥ Paul Scherrer Institute (PSI), Laboratory for Femtochemistry, Forschungsstrasse 111, CH-5232 Villigen PSI, Switzerland; # National Cooperative for the Disposal of Radioactive Waste (NAGRA), Hardstrasse 73, CH-543 Wettingen, Switzerland

**Keywords:** MX80, backfill, residual oxygen, anaerobic corrosion, aerobic corrosion, sulfate-reducing bacteria (SRB), carbon steel, deep geological repository (DGR)

## Abstract

Deep geological repositories for the disposal of radioactive waste rely partly on the integrity of canisters and on the inhibition of microbial growth by the bentonite barrier for the effective isolation of the waste from the environment. Canister integrity can be compromised by the activity of sulfate-reducing bacteria (SRB) and by abiotic corrosion. Unexpected aerobic microbial growth and SRB inhibition under anoxic conditions were observed in bentonite during a recent long-term *in situ* experiment, which raised the possibility that residual O_2_ may delay anaerobic growth. Here, to investigate the role of O_2_, bentonite was equilibrated with 0, 21, or 100% O_2_, compacted to 1.25 g/cm^3^, and deployed in a borehole for 1.5 years. Analyses revealed that the higher the O_2_ concentration in bentonite, the greater the biomass and the more *Desulfatitalea* sp. dominates the SRB population. The thickest corrosion layer product of carbon steel was found in the 21% O_2_ case, reflecting ongoing aerobic and anaerobic processes. In contrast, the most extensive structural Fe­(III) reduction within montmorillonite was observed at 0% O_2_. These findings demonstrate that residual bentonite O_2_ shapes microbial activity and alters corrosion dynamics, highlighting the importance of accounting for oxygen during early repository evolution.

## Graphical abstract

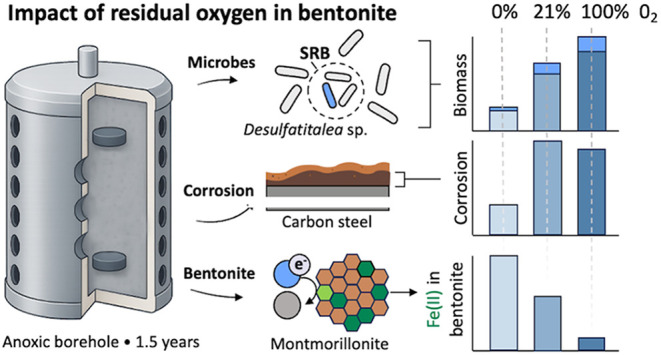

## Introduction

The long-term safety of deep geological repositories (DGRs) for radioactive waste relies on an engineered multibarrier system designed to effectively isolate the waste from the surface.^1,2^ The Swiss concept involves constructing tunnels in low-permeability rock hundreds of meters underground. In these tunnels, carbon steel (C-steel) canisters will be surrounded by a bentonite buffer.^3^ Wyoming bentonite MX80 is considered for its high montmorillonite content (up to 88 wt %) and its ability to retain radionuclides after canister breaching.^4^ Additionally, its swelling capacity upon contact with formation water gives it plasticity that reduces available pore space, thereby guaranteeing that the system remains diffusion-limited. This, together with high dry bentonite density (>1.45 g/cm^3^)^5^ in tunnels, is expected to effectively inhibit bacterial activity. Of particular concern for future DGRs is the activity of sulfate-reducing bacteria (SRB), which can use SO_4_^2–^ sourced from the dissolution of sulfate minerals (1.4 wt %)^6^ present in Wyoming bentonite^6^ and from porewater, and convert it to reduced S species, including HS^–^.^7^ This process can be fueled by a variety of electron donors,^8^ which in DGRs include gases such as CH_4_ and H_2_, and refractory solid organic matter (*ca* 0.1 wt % in Wyoming bentonite).^9–11^ HS^–^ can then diffuse through the buffer toward the canisters, potentially leading to their corrosion, a process referred to as microbially induced corrosion (MIC).^12,13^ MIC, alongside the abiotic aerobic and anaerobic corrosion processes expected post-DGR closure, can potentially compromise the long-term integrity of the canisters. In addition, SRB can also induce changes in the buffer either by production of HS^–^ or by direct enzymatic Fe­(III) reduction, causing the reduction of structural Fe­(III) in montmorillonite and other iron minerals in bentonite,^14–16^ such as goethite (0.6 wt %), lepidocrocite (0.9 wt %), ilmenite, hematite and magnetite (ca. 0.1 wt %).^6,17–26^ These changes may alter the porosity of the buffer and, in the case of montmorillonite, could lead to the formation of secondary Fe­(II)-bearing nonswelling clays, like Illite, ultimately impairing buffer safety functions.^27–29^

The activity of SRB in the repository is expected to be constrained by factors such as the lack of space and substrate availability, low water activity, high temperature, or irradiation,^13^ but also the presence of O_2_ introduced during excavation of the repository. It is expected that anoxic conditions, which are required for SRB activity will develop within a few weeks to months post closure, once all O_2_ has been depleted through processes like abiotic oxidation of Fe­(II) in mineral or metal components or the metabolic activity of aerobic heterotrophic bacteria.^30^ However, a recent long-term *in situ* incubation experiment under near-repository conditions demonstrated that, despite anoxic conditions, aerobic bacteria dominated the microbial community in Wyoming bentonite and there was no detectable contribution from SRB.^31^ This unexpected result suggests that, while bulk O_2_ has been removed, residual bentonite O_2_—potentially associated with microporosity—supports the growth of aerobes while either directly or indirectly inhibiting that of SRB. However, the impact of bentonite-associated O_2_ on the microbial community and on bentonite mineralogy remains unknown. Consequently, it is also unclear how the O_2_-induced changes in microbial communities and bentonite affect the corrosion of bentonite-embedded C-steel. This is critical, as C-steel is the material most frequently selected by waste management organizations^30^ and widely used in MIC^32–43^ studies.

To address this knowledge gap, we systematically varied the O_2_ concentration with which dry crushed Wyoming bentonite was equilibrated: 0, 21, and 100% O_2_ (v/v). The bentonite was compacted to a density allowing for microbial growth and deployed into an anoxic borehole for 1.5 years. With a combination of molecular biology, chemistry, and mineralogical tools, we aimed to assess the impact of trapped O_2_ on (1) microbial colonization, growth, and community composition, (2) mineralogical and chemical alterations in the bentonite backfill and (3) corrosion of carbon steel. The findings have significant implications for predicting postclosure conditions in waste deposition tunnels, offering new insights into microbial processes that may influence repository stability.

## Materials and Methods

### Experimental Setup

Twelve perforated stainless steel mini-modules (5.3 cm tall, 3.8 cm diameter) lined with stainless steel filters filled with 1.25 g/cm^3^ Wyoming MX-80 bentonite were placed into a larger perforated stainless steel module (25 cm tall, 12.6 cm diameter; Figure S1). The assembly was then incubated *in situ* for 1.5 years in an anoxic borehole at the Mont Terri Underground Rock Laboratory (URL) in Switzerland. The perforation allowed for Opalinus clay porewater infiltration. Prior to mini-module assembly, dry bentonite was ground to <3 mm and equilibrated for at least one year in (a) the absence of O_2_, by storage in 100% N_2_ atmosphere (hereafter, 0% O_2_), (b) atmospheric O_2_ (hereafter, 21% O_2_), by exposure to normal atmosphere, or (c) 100% O_2_. The varying O_2_ content in bentonite was confirmed in a parallel batch desorption experiment and quantified using a FireSting-O_2_ probe, showing that despite a year-long equilibration, traces of O_2_ could still be detected in 0% O_2_ sample (Supporting Information, Figure S2). In addition to the three treatments varying in O_2_ concentrations, γ-irradiated (see SI) bentonite equilibrated with atmospheric O_2_ (hereafter, 21%-S O_2_) was included as a sterile control. Each treatment involved three independent mini-modules (true replicates) and contained two coupons (24 coupons in total; 1.2 cm diameter, 0.3 cm thick; Amentum U.K.; Figure S3), out of which one per module (12 in total) was used for the carbon steel (C-steel) analysis. Before the assembly, all coupons were precleaned to remove any initial corrosion products (SI)

### Module Retrieval and Bentonite Sectioning

After removal from the borehole, the modules were packed under anoxic conditions (100% N_2_), transported to the laboratory, and stored for a week at 4 °C until processing. Next, on a bench and under continuous sterile N_2_ flow, using a sterile lever press, porewater-saturated bentonite cores were removed from mini-modules and transported into an anoxic glovebox (100% N_2_) and cut into three transversal sections of 1.5–2.0 cm thickness (see SI). The top and bottom sections of the core, containing coupons embedded in clay, were either resin-embedded (see SI) or the coupons were separated from clay and used for direct analysis of corrosion. The middle section (without the coupon) was subsampled to collect the inner part (2.51 cm diameter) and outer layer (a ring 1.29 cm thick). Samples for gDNA enumeration and sequencing were preserved at −20 °C, and air- or freeze-dried for mineralogical characterization. More details in the SI.

### DNA Extraction and Quantification

DNA was extracted using previously published methods^44,45^ following a modified protocol of DNeasy PowerMax Soil Kit (QIAGEN NV, Venlo, The Netherlands). Bentonite DNA was additionally purified by using 10 μL InvitrogenLinear Acrylamide (5 mg/mL), 0.1 volume of 5 M NaCl and 2 volumes of isopropanol. The air-dried DNA pellets were resuspended in 40 μL elution buffer provided in the DNeasy PowerMax Soil Kit and stored at −20 °C. Extracted DNA was then quantified using an Invitrogen Qubit 2.0 fluorometer (see SI). For quantification of bacterial 16S rRNA gene copy numbers, quantitative PCR (qPCR) was performed using the MYRA robotic system and a MIC qPCR Cycler (both BioMolecular Systems, Australia). Details on DNA extraction, qPCR reaction and primer pair sequences are in the SI.

### Sequencing and Bioinformatics

The full-length 16S rRNA gene (V1–V9) amplicon libraries were prepared following the standard procedure from Pacific Biosciences of California, Inc. (aka PacBio) and sequenced using the Sequel II system. Raw reads were quality-checked using FASTQC (v0.11.9).^46^ Adapter trimming, quality filtering, dereplication, error modeling, and amplicon sequence variant (ASV) inference were conducted using DADA2 (v1.30.0).^47^ Taxonomic assignment was performed using the RDP naive Bayesian classifier against the SILVA SSURef database (v138).^48,49^ All analyses were run on the EPFL high-performance computing cluster using SLURM (v23.11.10) and Apptainer (v1.2.5).^50,51^

### HCl and HF Fe Extraction

Fe was extracted from freeze-dried powdered outer layers of cores and reference materials, which included bentonite (MX80), γ-irradiated Wyoming bentonite (MX80-S), and chemically reduced Wyoming bentonite (MX80_red_). The digestion was performed using HCl and HF acids, with 0.5 M HCl used to quantify readily available Fe, including adsorbed and solid-phase Fe­(II) (e.g., siderite); with 6 M HCl to access more crystalline phases (e.g., magnetite); and with HF to quantify total Fe. The digestions were conducted in triplicate, and additional information on the digestion, analysis and chemical reduction of the clay are in the SI.

### ^57^Fe Mössbauer Spectroscopy

Mössbauer spectra collection was performed on freeze-dried powdered outer layers of cores and reference materials at 77 K using a close-cycle cryostat with a constant acceleration drive system (WissEL) in transmission mode with a ^57^Co/Rh source. Spectral analysis was carried out using Recoil fitting software (University of Ottawa) and the Voigt-Based Fitting (VBF) routine.^52^ More details are available in the SI and Table S2.

### X-ray Diffractometry (XRD)

was performed on freeze-dried powdered outer layers of cores and reference materials. Nonoriented bulk samples were directly analyzed, while oriented mounts were processed to prepare air-dried, glycolated and heated (550 °C; 1.5 h) samples (SI). XRD was performed using X’Pert MPD with X’Celerate equipped with a Cu-anode (Cu Kα radiation, λ = 0.15406 nm). The resulting diffractograms were analyzed with Match! (version 3.6.2.121) and Crystallography Open Database (ver. COD-Inorg REV248644 2020.03.03).

### X-ray Fluorescence (XRF) analysis

Maps of silicon (Si), sulfur (S), and iron (Fe; all K-edge) in bentonite and on the bentonite–coupon interface were obtained with an EDAX Orbis PCMicro EDXRF analyzer system (AMETEK Inc., Berwyn, Pennsylvania, U.S.A.), equipped with a Rh microfocus X-ray tube at 12 and 20 keV acceleration and 0.9–1 mA current with 30 μm polycap optics and an Apollo XRF-ML50 Silicon Drift Detector. The spectra were recorded for 100–500 ms at each spot with a step size of 30 μm.

### Bentonite Water Content

Bentonite water content and dry density were based on the wet weight loss after 24 h of drying at 105 °C, following the ISO procedure^53^ (calculation formula in SI). The dry weight of bentonite was used to normalize microbial and chemical data.

### Corrosion Analysis

The bentonite–coupon interface and the thickness of the corrosion product layer (CPL) were investigated using a Laser Confocal Optical Microscope (Keyence, VK-X200) with a Nikon objective (20 × 0.46, OFN25, WD-3.1 mm) in a depth composition mode. To calculate the CPL thickness, a minimum of 3 images at 20× and at least 30 measurements of CPL thicknesses were taken and analyzed by ImageJ. A Renishaw inViva Confocal Raman microscope, with a 532 nm laser and a 50X long-distance lens at optimum laser power in the range of 20 mW, was used to analyze the composition of CPL.

### Mass Loss and Corrosion Rates

The C-steel coupon mass was measured before the experiment and after removing corrosion products accumulated during incubation in the borehole. Details, together with the corrosion rate (CR) formula, are in SI.

### Statistical Analysis

ANOVA at α 0.05 determined significant treatment differences. For significant differences, pairwise *t* tests were performed with α 0.0083, adjusted via the Bonferroni correction method.

## Results and Discussion

### Microbial Growth in Bentonite and Adaptation to Oxygen

Quantification of microbial 16S rRNA gene copies confirmed growth in both the inner and outer parts of the bentonite core (Figure 1A), with most of the growth happening in the outer layers. Significant differences (*p* < 0.0083) were observed in outer core growth between 0% O_2_ and 21%-S O_2_, 0% O_2_ and 21% O_2_, and 21%-S O_2_ and 21% O_2_. On average, the least growth in the outer core could be quantified in the 0% O_2_ treatment (mean: 4.16 × 10^05^ copies/gdw), while the most was found in the 100% O_2_ case (mean: 1.93 × 10^06^ copies/gdw).

**Figure 1:**
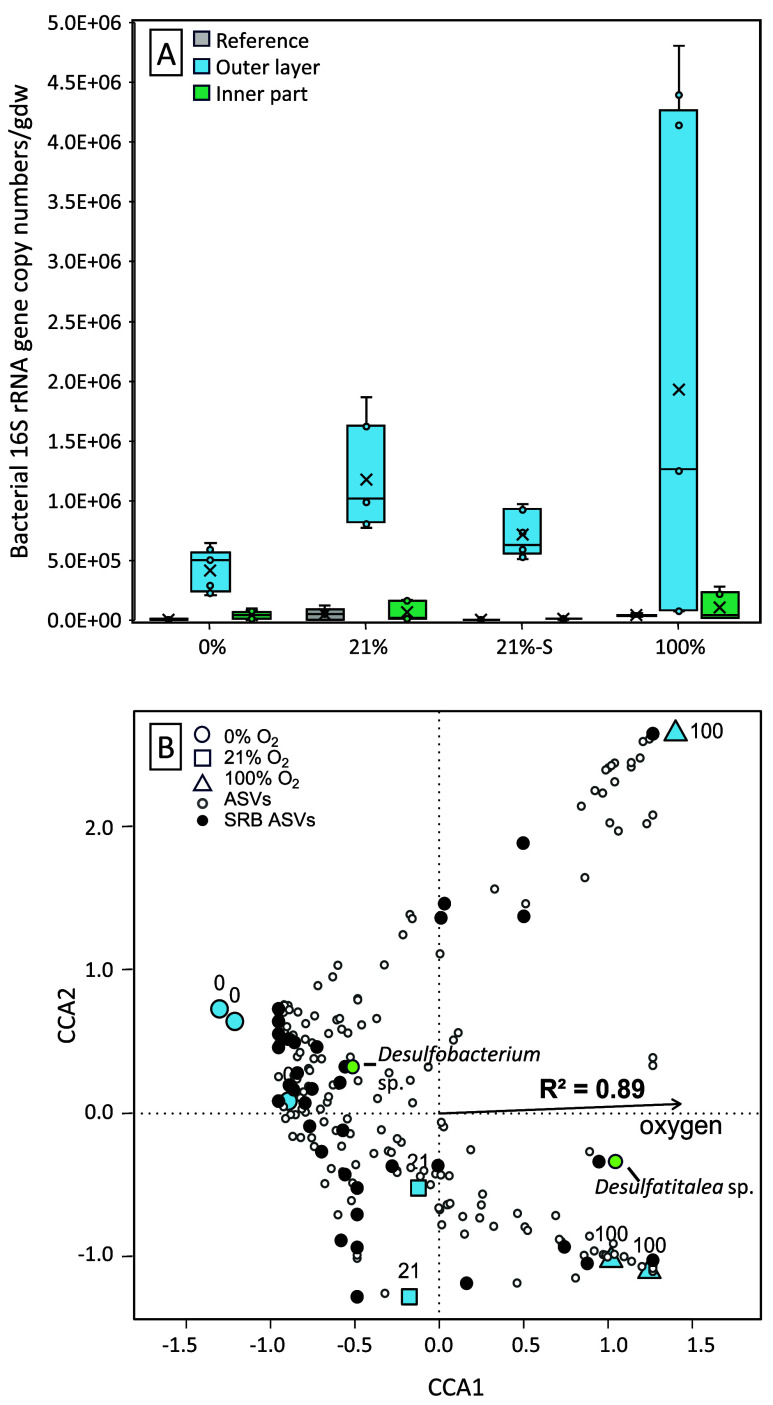
(A) 16S rRNA gene V4 region amplicon copy numbers in bentonite before deployment (gray) and after 1.5 years *in situ* incubation. Data differentiate gene copy numbers in the outer (green) and inner (blue) bentonite cores. The horizontal line in boxes indicates the median. “*X*” marks the mean. Whiskers show minimum and maximum values outside the first and third quartiles. All results were standardized to the dry weight of bentonite. (B) Canonical Correspondence Analysis (CCA) of microbial communities in the outer layer of the bentonite core, showing variation in relation to oxygen concentration with which bentonite was equilibrated before the deployment. Blue shapes represent individual samples in reduced-dimensional space according to community composition: 0% O_2_ (circles), 21% O_2_ (squares), and 100% O_2_ (triangles). Each small circle represents an ASV; sulfate-reducing bacteria (SRB)-related ASVs are shown in black, with the two most abundant SRBs labeled and marked in neon green. The arrow indicates the direction and strength of the correlation between O_2_ concentration and community composition.

Next, full-length 16S rRNA gene sequencing was used to determine microbial taxonomy of borehole and bentonite communities. (Figure S4). The list of all taxa was then examined for microbes typically associated with sulfate reduction. The borehole water consortium was dominated by SRB (41.89 ± 5.61% total relative abundance), with the most abundant ASVs being *Desulfobacterium* sp. (31.63 ± 0.85%) and *Desulfatitalea* sp. (6.39 ± 6.22; Figure S5). The rest of the SRB population consisted of *Desulfallas-Sporotomaculum* (0.76 ± 0.18%), and another 110 ASVs. After SRB-associated genera, the other most abundant genus was *Dethiobacter* sp. (27.12 ± 3.81%) and two *Pseudomonas* spp. *Pseudomonas xanthomarina* (*P. xanthomarina*) (19.83 ± 6.71%) was previously reported in Opalinus Clay,^54^ while *Pseudomonas stutzeri* (*P. stutzeri*) (1.17 ± 0.12%), a common facultative anaerobic bacterium, was previously found in compacted Wyoming bentonite in two long-term *in situ* incubation studies.^31,44^

The bentonite outer layer samples showed high similarity to borehole communities with a few striking differences (Figure S5). First, the *Pseudomonas* spp. in bentonite had much higher abundance than in porewater, representing, on average, 55.41 ± 9.45% of the population. Second, *Dethiobacter* sp., being the second most abundant microorganism in borehole water, became less abundant in bentonite samples to merely 1.0 ± 1.37%. Third, the overall SRB relative abundance in the bentonite outer layer was, on average, lower than in borehole water and depended on the bentonite O_2_ equilibration concentration. Moreover, the relative abundance of the two most abundant SRB changed across treatments: *Desulfatitalea* sp. was the most abundant at 100% O_2_, with 18.63 ± 6.3 relative abundance vs 0.94 ± 1.01 at 0% O_2_. Conversely, *Desulfobacterium* sp. had higher relative abundance at 0% O_2,_ at 18.90 ± 8.50 vs 3.98 ± 3.12 at 100% O_2_.

To investigate further the relationship between microbial diversity and O_2_ concentration, a Canonical Correspondence Analysis (CCA) was run on ASV counts from the outer part of bentonite cores (Figure 1B). Almost all the separation among samples occurred along the O_2_ gradient (CCA1 axis). Many of the ASVs identified in 0% O_2_ samples segregate from those at 100% O_2_, indicating that O_2_ persisting in bentonite had a strong impact on structuring the communities in our experiment. Microorganisms associated with known SRB (black circles) preferred but were not strictly limited to anoxia. Most SRB were found left of the zero line on the CCA1 axis, supporting their anaerobic niche. However, a subset of SRB-associated ASVs plotted near/above zero, including the most abundant SRB, *Desulfatitalea* sp., indicating a possible adaptation of some SRB to growth in the presence of residual O_2_.

A similar separation of ASVs depending on O_2_ was observed for the inner core samples (Figure S6). However, these microbial communities showed high variability among replicates, most likely due to bias related to overall very low cell numbers, as demonstrated by the low number of gene copies and bentonite heterogeneity (Figures 1A and S4). The community was then manually screened for ASVs commonly associated with taxa involved in processes related to bentonite reduction or C-steel corrosion, such as SO_4_^2–^ reduction, Fe­(III) reduction, Fe­(II) oxidation, and H_2_ oxidation (Figure S7). The latter pathway can be coupled to SO_4_^2–^ or Fe­(III) reduction by autotrophic or/and mixotrophic bacteria under anoxic conditions. The SRB *Desulfatitalea* spp. was the most abundant in 100% O_2_ samples. In addition, *Thiobacillus* spp., potentially involved in S oxidation and Fe­(III) reduction were present in 0 and 21% O_2_ samples, while potential H_2_ oxidizers, e.g., *Hydrogenophaga* sp., were present under all conditions. Finally, *Acidovorax* sp., a potential Fe­(II)-oxidizer, was found in 0% O_2_ bentonite.

The data demonstrate that microbial communities grew inside compacted (1.25 g/cm^3^) bentonite, and their structure and abundance were shaped both by varying O_2_ availability and the input of borehole bacteria. In all samples, 16S rRNA gene copy numbers in the outer layer increased relative to as-received bentonite, were consistently higher than in the inner core (Figure 1A), and showed a positive correlation with initial O_2_ content (Figure S8). The difference in growth between the outer and inner layers was particularly noticeable in the 21%-S O_2_ sample, where γ sterilization had effectively inactivated most of the indigenous microorganisms before deployment. After incubation, gene copy numbers within the sterile core showed no significant increase compared to the sterilized as-received bentonite, whereas the outer layer supported abundant communities. This pattern indicates that the outer-layer growth was largely due to colonization by porewater microorganisms, with O_2_ further controlling the extent of proliferation. However, the contribution of bentonite taxa, whose growth could have been promoted due to a potentially lower bentonite density in the outer versus inner layers, or grow of the porewater microbes on the very surface of the bentonite core (and not within), cannot be excluded.

The colonization can be further supported by the taxonomy and abundance of the outer bentonite communities overlapping with but being distinct from, those of borehole water (Figures S4–S5). This is consistent with selective pressures favoring certain porewater taxa such as *P. stutzeri* and *Dethiobacter* sp. for growth inside bentonite while suppressing others like *Dethiobacter sp.*. Similar differentiation between borehole water and compacted MX80 has been reported before,^55^ with *Clostridia* spp. growing in clay, *Desulfovibrio aespoeensis* (*D. aespoeensis*) in water, while *Sedimentibacter* and *Desulfosporosinus* were found in both, further supporting that some borehole bacteria may prefer bentonite as their habitat and thus, colonize it.

Although our study cannot accurately determine the depth of the colonization front due to the limited sampling spatial resolution, it is likely to be within a centimeter range. This is supported by an observation that in two previous 5.5-year-long incubation studies of bentonite in different rock types, but using the same sampling approach, where the outermost bentonite surface layer (up to centimeters thick) was discarded, reported little or no borehole bacteria in bentonite.^31,56^ Together, this provides a basis for a mechanistic interpretation, suggesting that colonization does happen, but it is restricted to a short time and distance. This window occurs during the saturation of dry bentonite, when advective flow and suction transport porewater containing cells. It is then followed by swelling, which closes the pore space and, combined with high bentonite density (1.25 g/cm^3^), prevents microbial mobility and suppresses growth.^31^

Finally, this study demonstrates the adaptation of some SRB to the presence of O_2_ in bentonite.^58^ More specifically, while most detected SRB species preferred bentonite with lower or no O_2_, some favored growth in bentonite equilibrated with 100% O_2_ (Figure 1B). A striking example was the abundance of *Desulfatitalea* sp., whose closely related genera were reported to inhabit deep subsurface environments (AB237684, query cover 100%, identity: 97.15%, Figure S9) and anaerobic niches.^57,58^ Importantly, SRB are generally strict anaerobes,^59^ with the few known to be O_2_-resistant or microaerophilic being affiliated with *Desulfovibrio* spp.^39,41–43,60^ In our study, *Desulfatitalea* sp. was found to be the most abundant based on both relative and absolute 16S rRNA gene copy numbers (Figures S5–S6) in cores equilibrated with 100% O_2_ compared to other bentonite samples and to porewater. We interpret this positive correlation with increasing O_2_ content (Figure 1B) as evidence that *Desulfatitalea* sp. was metabolically active and may possess previously unreported O_2_-resistant traits, allowing it to outcompete less O_2_-tolerant bacteria. In addition, it could have the ability to perform aerobic respiration, as documented for some SRB, including *Desulfurovibrio* species.^60^ A third explanation is that the growth of *Desulfatitalea* sp. and other SRB in bentonite equilibrated with 100% O_2_ is supported by metabolites (e.g., organic compounds) produced by aerobic microorganisms that potentially are the first ones to grow in this system, using residual O_2._ The provision of organic carbon by aerobic bacteria fueling sulfide production by SRB was previously observed in sulfidic spring microbial mats.^61^ These metabolites may have facilitated SRB growth that commenced after the depletion of O_2_.

### Solid Phase Analysis

To access different Fe pools in the outer core samples, from readily available Fe to structural Fe in clays (for details see SI), extractions using HF or two HCl concentrations were used. The 0.5 M HCl extraction only yielded Fe­(II), and the concentration was similar across all samples (including the as-received MX80), an average of 1.95 ± 0.32 mg/gdw of Fe­(II) (Figure 2 and Table S1). The 6 M HCl extraction was used to access the more crystalline phases (e.g., magnetite^62,63^ ) and yielded an average of 7.00 ± 0.47 mg/gdw of total Fe concentration from all the samples, except 0% O_2_ samples, which yielded a higher concentration (12.81 mg/gdw). Interestingly, a similar 6 M HCl-extractable total Fe (14.07–14.49 mg/gdw) can be obtained from MX80 chemically reduced by Na_2_S or Na_2_S_2_O_4_ (Table S1). Finally, HF was used for the quantification of total Fe and total Fe­(II) in the samples. The results showed that there was no significant difference in total Fe content between the samples (pairwise *t* tests, *p* < 0.5), on average reaching 23.38 ± 1.67 mg/gdw, corresponding to 2.34 ± 0.17 wt %, allowing for the Fe data to be compared and presented in terms of relative values (Figure 2; absolute values in Table S1). Fe­(II) content varied between the samples, with the lowest content in the as-received MX80 yielding 10.10% of total Fe, while samples incubated in the borehole followed a general trend: the higher the O_2_ content in the atmosphere before incubation, the less Fe­(II) was present. 100% O_2_ samples had 22.78% Fe­(II), whereas 21% O_2_ samples harbored 32.48% Fe­(II), and 21%-S O_2_ samples, 22.67% Fe­(II). Finally, the highest Fe­(II) concentration was found in 0% O_2_ bentonite, yielding 43.22% Fe­(II).

**Figure 2:**
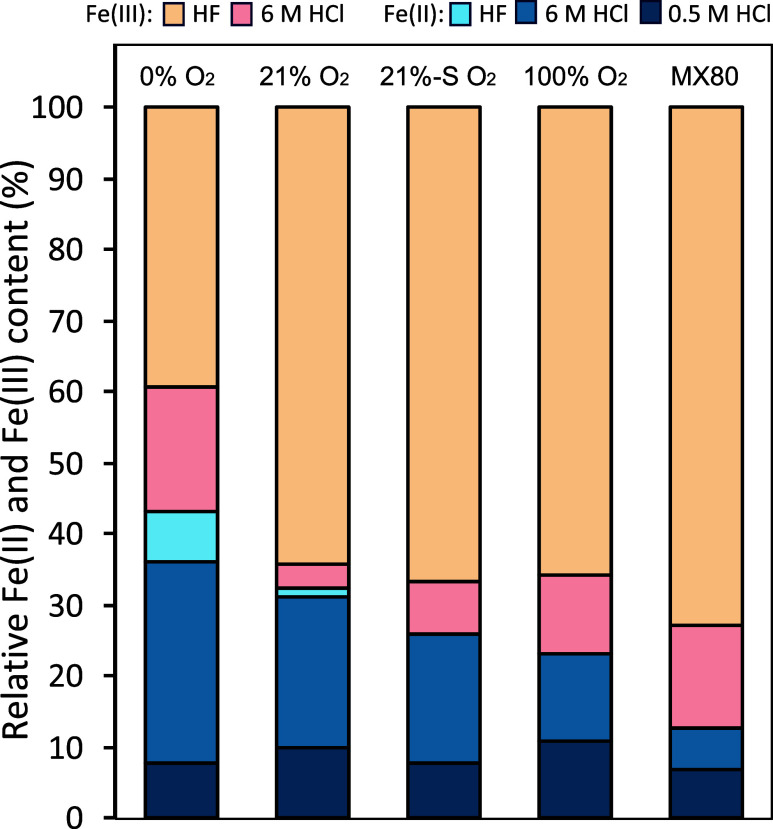
Fe­(II) (blue) and Fe­(III) (orange) extracted from bentonite outer cores after 1.5 years in a borehole, using 0.5 M HCl (readily available Fe), 6 M HCl (more crystalline phases), and HF (total Fe). Results were standardized to g of Fe per dry weight of bentonite. HF-digested Fe was used as total Fe for calculating relative abundance of each fraction.

X-ray diffraction (XRD) unveiled montmorillonite as a main clay component with a minor contribution of Illite in as-received MX80 (Figures 3A and S10). Bentonite also contained feldspars (xAl­(Al,Si)_3_O_8_: orthoclase, sanidine), quartz (SiO_2_), gypsum (CaSO_4_), calcite (CaCO_3_), goethite (α-FeO­(OH)) and pyrite (FeS_2_), commonly found in MX80 (e.g,^6^). After *in situ* incubation, new phases like magnetite (Fe_3_O_4_) and siderite (FeCO_3_) were formed only in the 0% O_2_ sample (Figure S10). A stepwise procedure including glycolating and heating the oriented mounts showed no montmorillonite alteration, such as formation of nonswelling clays (Figure S11). Next, Mössbauer spectroscopy was used to follow the fate of Fe in mineral phases. The spectra exhibited two Fe­(II) and two Fe­(III) doublets, most likely stemming from octahedrally coordinated Fe in montmorillonite, since all other Fe-bearing phases had too low relative content to generate a detectable ^57^Fe signal. The summed results confirmed the HF-extraction data showing an increase in Fe­(II) and a decrease in Fe­(III) content as the O_2_ content preincubation decreases (Table S3). The as-received MX80 showed an overall Fe­(III) content of 64.6%, while the Fe­(III) in MX80_red_ decreased to 30.6%. The 100% O_2_ sample showed up to 68.65% Fe­(III), while the 0% O_2_ and 21% O_2_ samples showed 48.6% and 55.7% Fe­(III), respectively. The 21%-S O_2_ sample exhibited 65.5% Fe­(III), 10% more than its nonsterile equivalent (Figures 3, S12 and Table S3).

**Figure 3:**
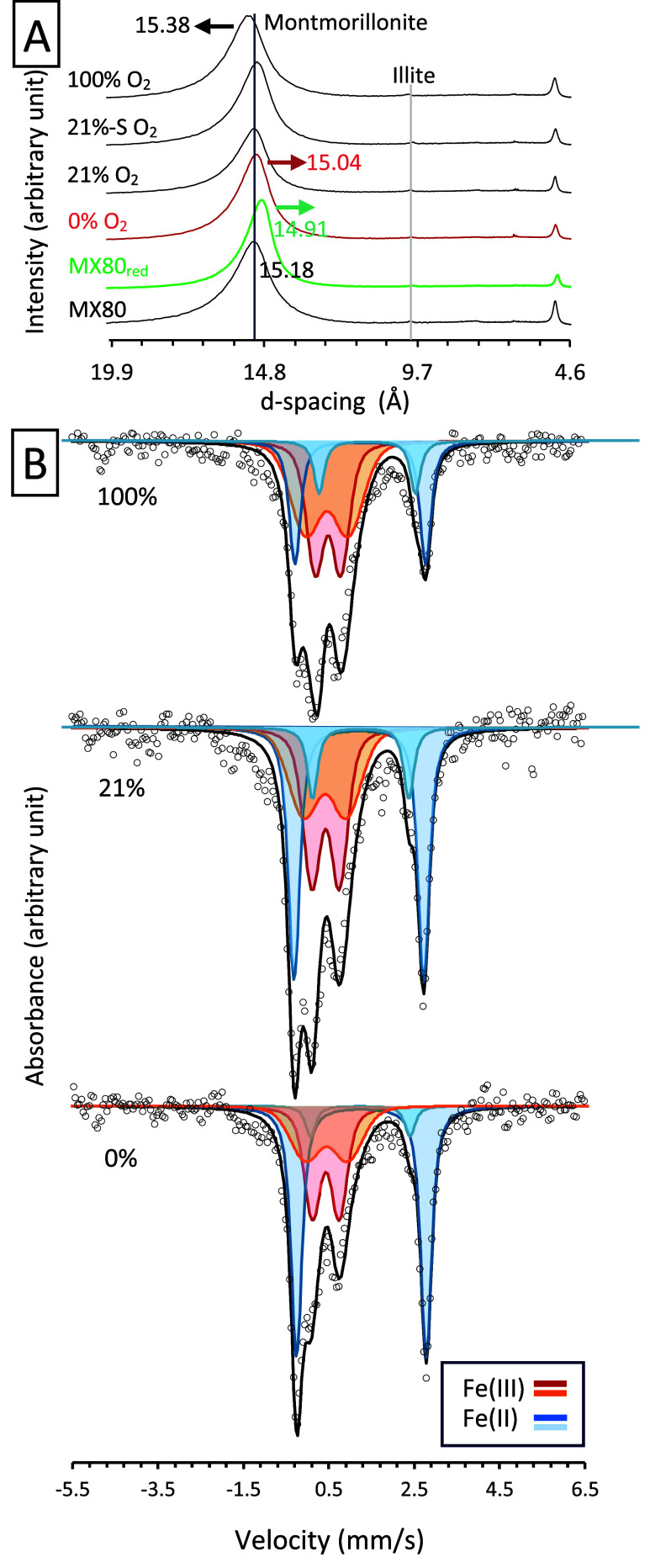
Mineralogical characterization of reference materials (MX80 and MX80_red_) and samples after *in situ* incubation. (A) XRD patterns of randomly oriented bulk powders. Chemically reduced MX80 (MX80_red_) is marked with green. The vertical lines represent the position of the basal montmorillonite and Illite reflections. Numbers above the patterns show *d*-spacing values for montmorillonite. (B) Mössbauer spectra (collected at 77 K) of 0% O_2_, 21% O_2_, and 100% O_2_ samples after the *in situ* incubation, showing an increase in the plot area related to the relative Fe­(II) content (blue doublets) and a decrease in the area representing Fe­(III) (orange-red doublets) with decreasing O_2_. Empty circles represent raw data; the black line shows the fitted spectrum.

Micro X-ray Fluorescence (μXRF) analysis was used to map the distribution of Fe (Figure S13) and S (Figure 4) in the bentonite cores. Fe was used as an indicator of potential Fe phases, such as pyrite (FeS_2_), siderite (FeCO_3_) and iron sulfides (FeS_*x*_) and Fe-containing clays (approximate montmorillonite formula (Na,K,Ca)_0.3_(Al,Mg,Fe,Ti)_2_Si_4_O_10_(OH)_2_·nH_2_O). Fe showed uniform distribution patterns reflecting the distribution of clays. Sulfur (S) was used to trace sulfate stemming from bentonite-derived gypsum (CaSO_4_·2H_2_O) or FeS_*x*_, present in the as-received bentonite, or formed during the *in situ* incubation upon reduction of sulfate derived from porewater. S varied between samples, forming S-enriched bands of differing thicknesses (Figures 4 and S13). The 0% O_2_ condition had the thickest layer (5.60 ± 0.43 mm), while for 100% O_2_ it measured only 0.46 ± 0.1 mm. In summary, the less O_2_, the thicker the S-enriched band in the outer layer, while Fe and Si remain evenly distributed across samples.

**Figure 4:**
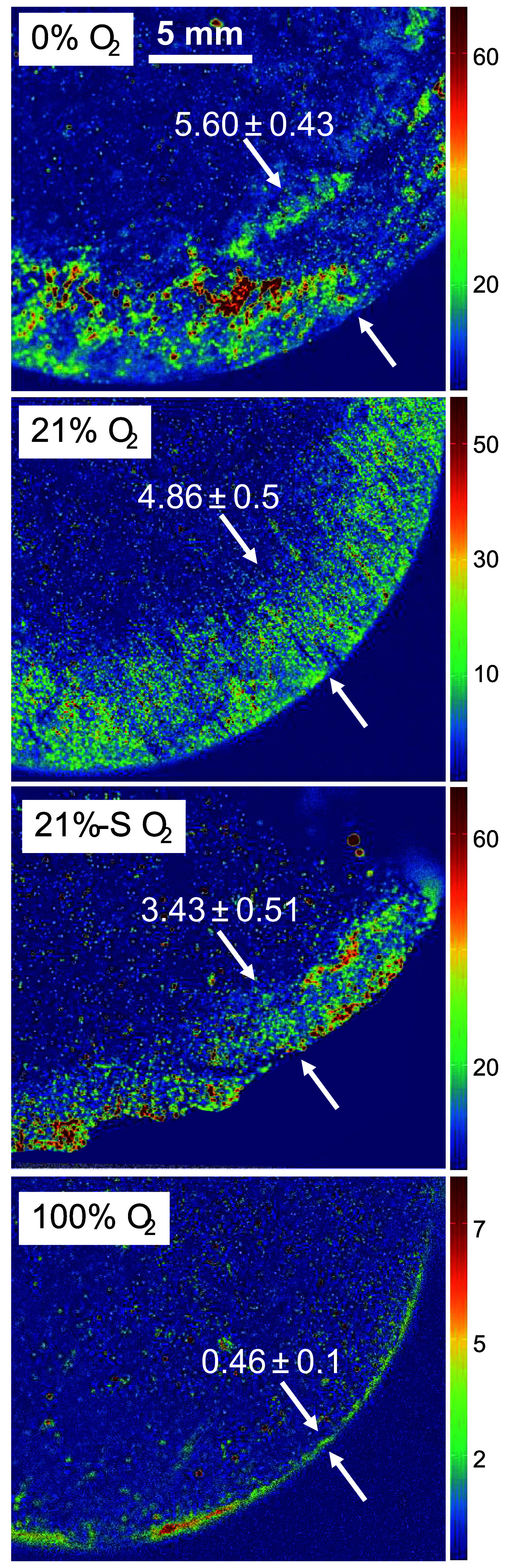
XRF elemental distribution maps of sulfur within the outer layer of the bentonite cores. Horizontal panels represent 0% O_2_, 21%-S O_2_, 21% O_2_ and 100% O_2_ bentonite treatments. The white arrows show the area enriched in S, reflecting the sulfate reduction front in the bentonite core in contact with sulfate-containing borehole water. Numbers show the thickness (*n* > 30) of the band in mm.

The most Fe­(III) reduction was observed in the 0% O_2_ and the least in the 100% O_2_ condition, providing direct evidence that the amount of associated O_2_ is a controlling factor for the extent of structural Fe­(III) reduction in bentonite (Figures 2 and 3). Furthermore, structural Fe­(III) reduction in clay can be supported by three pieces of evidence. First, structural Fe in montmorillonite accounts for >90% of the total Fe in MX80, while other Fe-bearing minerals such as (oxyhydr)­oxides or pyrite account for ≤5% of total Fe.^64^ In 0% O_2_, Fe­(II) increased by 33.12%, compared to as-received MX80. Therefore, montmorillonite structural Fe­(III) reduction must account for a large part of this increase. Second, the XRD data point to a shift of the basal smectite peak (001) to lower *d*-spacing values, consistent with the shift observed for chemically reduced montmorillonite (MX80_red_). Third, Mössbauer spectra demonstrated the reduction of octahedral Fe­(III) represented by two doublets identified as corresponding to montmorillonite, while the signal from other phases was not detected due to their low relative contribution. These findings indicate that after the depletion of sorbed O_2_ due to (i) the desorption, dissolution and diffusion of O_2_ outward after contact with anoxic borehole water, (ii) abiotic mineral oxidation, and (iii) biotic oxygen reduction, Fe­(III) reduction was induced by microbial activity.

In this system, bentonite Fe­(III) can be reduced by Fe_aq_^2+^ from anaerobic corrosion of C-steel, by the activity of SRB producing HS^–^, and/or via direct enzymatic Fe­(III) reduction by bacteria such as *Dethiobacter* sp. This bacterium was previously implicated in sulfur species disproportionation and thiosulfate reduction with H_2_, as well as enzymatic Fe­(III) reduction.^65,66^ Additionally, Fe­(III) reduction could be mediated by *Thiobacillus* spp., some species of which are known as Fe­(III)-reducers.^67^ Microbially mediated Fe­(III) reduction was evidenced by HF-extracted Fe­(II) content postincubation for the 21% O_2_ and 21%-S O_2_ bentonite samples. For 21% O_2_ samples, the Fe­(II)/Fe­(III) ratio was 45%, higher than that of the sterile one (ratio of 0.29), underscoring the role of microbial activity (Table S1). Further, the relative difference in Fe­(II) content attributable to microbially driven Fe­(III) reduction is most likely underestimated because a large portion of Fe­(II) measured in the sterile control comes from reduction caused by γ irradiation (50 kGy total dose). A total dose of 40–60 kGy of radiation is expected to increase the Fe­(II)/Fe­(III) ratio in MX80 from ∼0.1 to ∼0.17–0.27 ^68^ and the ratio in this study (after *in situ* incubation) was 0.29 (Table S1), suggesting limited Fe­(III) reduction in the irradiated bentonite.

Altogether, microbial growth and activity within bentonite were supported by a difference in microbial cell numbers, where an increase in abundance was observed across all samples. In addition, at 0% O_2_, the growth was likely partly supported by metabolic processes involving S and Fe chemical species as electron acceptors, as the traces of O_2_ that could be initially present were most likely rapidly depleted.

### Corrosion Analysis

Cross sections of the bentonite–coupon interface for all conditions revealed the following zonation based on the microscopic observations combined with elemental distribution collected with XRF (Figure S14): (1) the C-steel coupon, (2) a corrosion product layer (CPL) covering the C-steel surface, (3) an altered zone within bentonite, and (4) unaltered bentonite. Across the samples, the thickness of the CPL and the altered zone differed significantly (Table S4) depending on the treatment. Among the unsterilized bentonite samples, the thinnest CPL thickness was observed in 0% O_2_ samples (38.1 ± 19.2 μm) while coupons placed within 21% O_2_ or 100% O_2_ bentonite were significantly thicker (*p* < 0.05): 121.5 ± 79.7 μm and 96.5 ± 33.9 μm, respectively. These values exceed those reported for the coupons in the previous *in situ* experiments in the same borehole.^35^ However, the incubation time, dimensions of the modules, resin embedding method, and CPL thickness analysis method differed. The thickness of the altered zone showed an inverse trend, with the thickest zone measured for coupons in 0% O_2_ bentonite (1.5 ± 0.2 mm), while significantly lower values (*p* < 0.05) were measured for 21% O_2_ (nonsterile), and 100% O_2_ bentonite (1.2 ± 0.1 mm and 1.2 ± 0.2 mm, respectively). The thickness measured for the 21% O_2_ case was in agreement with previous studies, including the thickness of the FeOOH layer model predicted by a numerical simulation.^69^ When comparing the unsterilized and the γ-sterilized bentonite samples, the sterilized sample exhibited significantly (*p* < 0.05) thinner CPL but thicker altered zone than the sample containing unsterilized bentonite. Overall, in all comparisons between treatments, the following could be observed: the thicker the CPL, the thinner the altered zone layer, and vice versa (see SI for additional discussion). Next, mass loss was used to quantify the average corrosion rates (Table S4). The calculated corrosion rates showed similar values for 0, 21, and 21%-S O_2_ bentonite treatments: 2.50, 2.25, and 2.75 μm/year, in agreement with range of values published before,^35^ while 100% O_2_ exhibited nearly double the corrosion rate: 4.25 μm/year. μRaman on the corrosion layer showed the presence of siderite, magnetite and goethite as the main products in all treatments (Figure S15).

Progressive O_2_ depletion is anticipated in the repository postclosure. This redox evolution will directly affect corrosion processes, including MIC, resulting in the formation of, first, oxic and then anoxic corrosion products on the canister–bentonite interface. When comparing 100% O_2_ bentonite, where the activity of sulfate-reducing bacteria (SRB) was expected to be suppressed, to 0% O_2_ bentonite, where SRB activity was expected to be enhanced, the CPL layer was significantly thicker in the former. Thus, the effects of abiotic O_2_-driven corrosion outweighed the potential role of microbially driven corrosion. Our data show that, over a short time frame, O_2_-dependent abiotic corrosion has a greater impact on canister stability than MIC occurring under anoxic DGR conditions. Given the inhibitory properties of high-density backfill (>1.45 g/cm^3^) and results from previous long-term (5.5 years) *in situ* incubation experiments,^31^ where microbial growth in compacted bentonite was shown to be limited, and SRB abundance insignificant,^31^ MIC is not expected to significantly contribute to the degradation of the canister, even after complete depletion of O_2._

The coexistence of reduced Fe­(II)-bearing mineral phases (magnetite, siderite, reduced montmorillonite), and newly formed oxidized phases (e.g., goethite) in the altered zone associated with the coupons indicates that redox conditions during the incubation shifted from oxidizing to reducing. This transition occurred in all samples, regardless of the initial amount of bentonite-associated O_2_, including 0% O_2_ bentonite, most likely due to the traces of O_2_ remaining in the bentonite (evidenced by the O_2_ dissolution experiment (Figure S2)).

### O_2_ Evolution in DGRs: A Conceptual Model

Combining previous observations of C-steel corrosion in compacted bentonite^32,70–73^ and the results from this study, the following model is proposed (Figures 5 and S16 for illustration). First, starting from the coupon, aerobic corrosion of C-steel is initiated because of contact with water in bentonite and porewater entering the pores of bentonite. This leads to the formation of Fe­(III) (oxyhydr)­oxides (hematite or goethite/lepidocrocite^74^). As the corrosion layer thickens, direct contact of the Fe^0^ in coupon with O_2_ and H_2_O progressively decreases, and the diffusion of O_2_ from bentonite slows down, because of the growing layer and gradual depletion of O_2_. Second, after O_2_ in the near vicinity of the coupon is depleted, anaerobic corrosion of the C-steel starts, leading to the generation of Fe_aq_^2+^, which reduces previously formed (oxyhydr)­oxides into magnetite, or forms siderite. The thickness of this CPL layer is dictated by the thickness of the primary (oxyhydr)­oxide layer. Remaining Fe_aq_^2+^ (not used in CPL formation) diffuses through the bentonite matrix and is oxidized by residual O_2_ in the clay, where it precipitates forming the altered zone. This model is supported by the altered zone being thicker in samples initially equilibrated with an atmosphere containing only trace amounts of O_2_ (i.e., 0% O_2_ bentonite, Figure S2) than those in 21 or 100% O_2_ samples. Indeed, a thinner altered zone is observed in the latter two, because most of the Fe_aq_^2+^ released from C-steel was incorporated into the CPL or oxidized by O_2_ in bentonite. Most of the Fe­(II) oxidation likely happens abiotically as microaerophilic Fe­(II)-oxidizing bacteria have not been identified in the samples, and other electron acceptors (e.g., nitrate) that could support *Acidovorax* sp., a putative Fe­(II)-oxidizer, are absent. In addition, the remaining Fe_aq_^2+^ that did not participate in the transformation or formation of new Fe­(II) phases will reduce Fe­(III) in minerals such as goethite or montmorillonite, as supported by the acid digestion data. Fe­(III) reduction in clays may lead to retaining Fe­(II) within the structure or reductive clay dissolution, causing the formation of Al–Si gels. Si in contact with Fe^2+^ may further form Fe­(II)-silicates, as previously suggested,^75^ but these were not identified in this study

**Figure 5:**
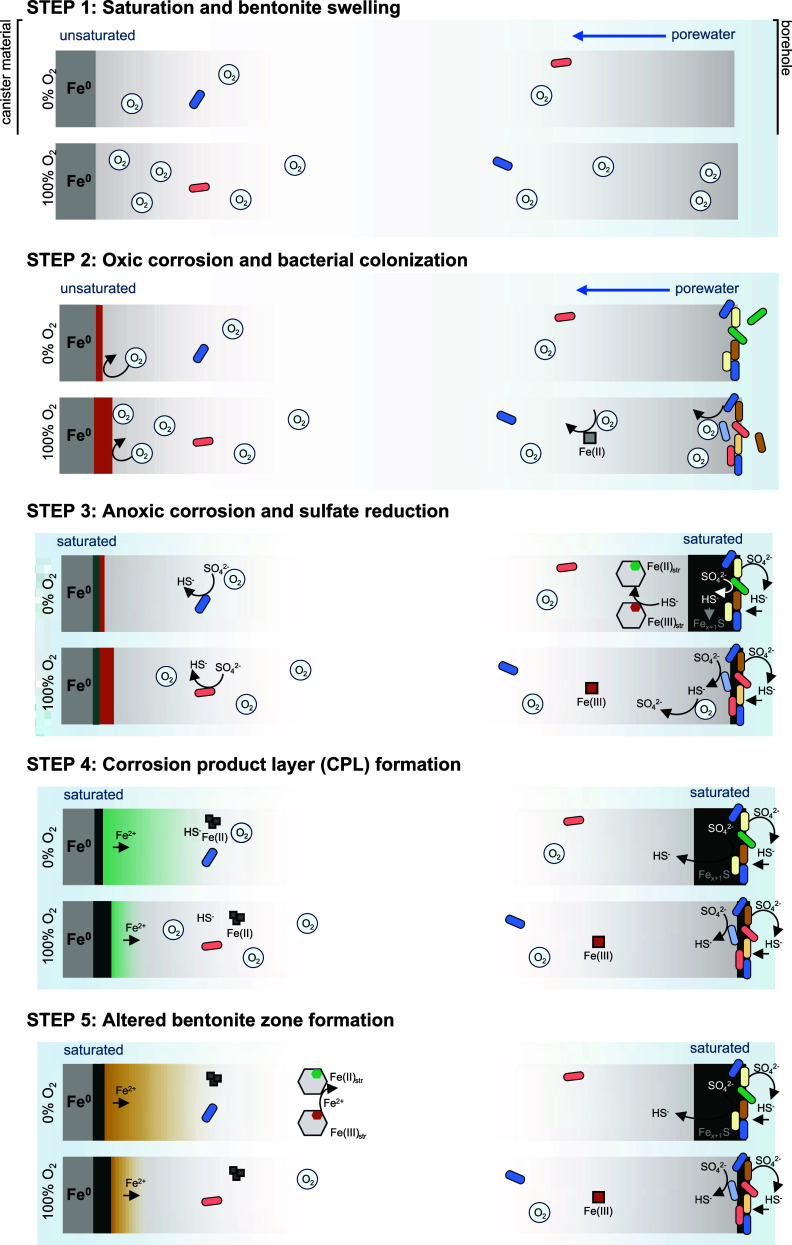
Conceptual model depicting the formation of corrosion products of carbon steel and the microbial activity within bentonite. For each step, the upper panel illustrates samples equilibrated at 0% O_2_, while the lower panel corresponds to samples equilibrated at 100% O_2_. The schematics show two regions: the near-field, next to the canister material (left), and the far-field, represented by the bentonite in direct contact with porewater and the Opalinus Clay host rock. An additional point-by-point description is available in Figure S16.

Redox changes at the bentonite–borehole water interface start from porewater entering the bentonite, leading to immediate dissolution of bentonite-absorbed O_2_, as demonstrated in the O_2_ dissolution experiment (Figure S2). Next, O_2_ decreases due to its diffusion into the anoxic water, microbial consumption mostly by borehole water bacteria such as *Pseudomonas* spp., and abiotic reactions such as the oxidation of Fe­(II) phases (e.g., pyrite) present in the as-received bentonite. Next, microbial sulfate reduction commences, leading to the formation of sulfide. This sulfide can reduce Fe­(III)-bearing minerals (e.g., montmorillonite), react with reduced Fe_aq_^2+^ (if present) precipitating iron sulfide minerals (e.g., mackinawite), or react with residual O_2_ to form intermediate valence sulfur species that could be further used by SRB. Thus, the initial bentonite O_2_ content controls the extent of Fe­(III) reduction, the S precipitation front, and the composition of the microbial community via dictating the composition of SRB or Fe­(III)-reducers.

### Environmental Implications

Our findings indicate that although future repository conditions are anoxic, the O_2_ that will be present initially and for a short period, will play a crucial role in controlling the abundance and composition of microbial communities, Fe­(III) reduction in bentonite, and C-steel corrosion. Here, the initial presence of O_2_ enhanced the growth of microorganisms within the bentonite. Surprisingly, this included SRB, particularly *Desulfatitalea* sp., even though this metabolic group is typically considered to be anaerobic. While results suggest microbial contribution to C-steel corrosion and alteration of bentonite, that contribution was less significant than abiotic reactions. Importantly, these findings were observed at a lower bentonite density (1.25 g/cm^3^) than the current minimum target density (1.45 g/cm^3^). Since dry density directly affects diffusion rates and microbial activity, higher-density scenarios in future repositories will further limit microbial-driven alteration, thereby reducing their overall impact. Thus, under the planned repository conditions, microbial processes are unlikely to pose a significant long-term threat to canister integrity or geochemical stability, despite the observed initial boost from O_2_.

## Supplementary Material



## Data Availability

Code and reproducible environments (Dockerfiles) for bioinformatic analysis are available here: http://github.com/nlmjacquemin/ICA6ANA2.

## Notes

Detailed information on methods; images showing the design and the assembly of the mini-modules; plot with O_2_ desorption data, plots showing sequencing results; additional plots showing XRD patterns of bulk and oriented bentonite samples; additional Mössbauer spectra of controls; additional confocal microscopy and XRF maps of bentonite and bentonite–coupon interface; μRaman spectra of CPL showing corrosion products; illustration of the conceptual model of corrosion products formation; tables with Fe extraction data, Mössbauer with fitting parameters, and coupon corrosion rates (PDF)
