# Supplementary material for: Impact of Oxygen Release from Bentonite on Microbial Activity, Mineralogy, and Steel Corrosion
Source: Environ Sci Technol. 2025 Nov 19;59(47):25368–79. doi: 10.1021/acs.est.5c08788 (PMC12676745; doi:10.1021/acs.est.5c08788)
Supplement: Supplementary file 1 [file es5c08788_si_001.pdf]

Supplementary information

Impact of oxygen release from bentonite backfill on microbial activity, bentonite mineralogy and canister corrosion

Natalia Jakus[1*], Pranav Vivek Kulkarni[2], Carolin L. Dreher[3], Sylvie Bruggmann[4], Daniel Grolimund[5], Andreas Kappler[3], Nikitas Diomidis[6], Stefano Mischler[2], Rizlan Bernier-Latmani[1]

[1]Environmental Microbiology Laboratory, École Polytechnique Fédérale de Lausanne (EPFL), Switzerland
[2]Tribology and Interfacial Chemistry Group, École Polytechnique Fédérale de Lausanne (EPFL), Switzerland
[3]Geomicrobiology Group, University of Tübingen, Germany
[4]Institute of Earth Sciences, University of Lausanne, Switzerland
[5]Paul Scherrer Institute (PSI), Laboratory for Femtochemistry, Switzerland
[6]National Cooperative for the Disposal of Radioactive Waste (NAGRA), Switzerland

*Corresponding author: natalia.jakus@epfl.ch

Number of pages: **22**
Number of figures: **16**
Number of tables: **5**

1. Supplementary methods

Cylinder materials. All materials used to manufacture parts that were used to host and deploy bentonite inside the borehole were made of two types of austenitic (stainless) steel. Modules and mini-modules parts, including cover, cylinder and screws were manufactured from 1.4301 (REMANIT-4301 according to EN 10 088-3 / DIN 17 440) while mesh filter manufactured from REMANIT-4404 according to EN 10 088-3 / DIN 17 440).

Gamma irradiation of bentonite. The gamma-irradiated bentonite served as a sterile control. For sterilization, Wyoming MX-80 bentonite was placed in an anoxic Schott bottle, flushed with N_2, and sealed with a rubber septum. A minimum dose of 50 kGy was applied using a Cobalt-60 source at Wood PLC in Harwell, Oxfordshire, UK, and stored in the sealed bottle until the module assembly. Note that even though gamma irradiation is the most effective sterilization method for rock powders and induces minimal changes, it can affect relevant bentonite properties, including Fe(III) reduction, water content, and carbon speciation. Consequently, redox processes, microbial activity, or diffusion rates can be affected. Thus, while it is the best control available, it is not perfect and, thus, does not allow for a complete comparison of the two materials (21% O_2 and 21%-S O_2).

Removal of the initial corrosion products from the coupon surface before emplacement. C-steel coupons underwent sequential washing steps using (1) 1.2 M HCl, (2) anoxic MQ, and (3) methanol to remove corrosion products. For each washing step, the coupons were placed in a flask with the solution and submerged in an ultrasonic bath for 10-30 min. The final washing step was followed by drying under a continuous flow of N_2, and the coupons were stored under anoxic conditions until the mini-modules were assembled.

The desorption batch experiment. The O_2 desorption capacity of bentonite upon contact with artificial porewater (modified APW, [1]) was confirmed in a separate O_2 dissolution experiment, in which the three bentonite powders (1 g each) were suspended in individual serum bottles containing 57 mL of anoxic APW (no gaseous headspace), and aqueous O_2 release was monitored (Fig S3) using a fiber-optic meter (FireSting-O_2, PyroScience GmbH, Aachen, Germany). Post-incubation O_2 was not measured because retrieval and processing involved multiple steps that could alter O_2 concentrations, including transport and storage under strictly anoxic conditions (risk of desorption), brief exposure to air (risk of sorption), and repeated vacuum–N_2 flushing cycles during transfer into an anoxic glovebox (risk of desorption). As reliable measurements could not be ensured under these conditions, O_2 concentrations were not determined.

Modules retrieval and bentonite sectioning. After incubation, the module containing twelve mini-modules was retrieved from the borehole and immediately transferred to the anoxic glovebox (100% N_2) available at the site (Niche MA, Mont Terri URL). In the glovebox (100% N_2), the mini-modules were removed from the module and packed into Mylar® bags. Bags were then removed from the glovebox, transported to the laboratory and stored at 4°C until processing. The bentonite cores were recovered from the mini-modules using a sterile lever press (in an ambient atmosphere) and sectioned under sterile and anoxic conditions (100% N_2 glovebox). Each core was cut into three slices (1.5-2.0 cm thickness); two containing the steel coupons and the middle one without a coupon (Fig S2). Maintaining anoxic conditions, the sections containing coupons were resin-embedded (SI) for corrosion characterization, while the middle sections of the cores were further sectioned using a sterile cylindrical tool to collect samples representing the inner part of the core (2.51 cm diameter) and the outer layer (a ring of 1.29 cm thickness), and preserved at -20° C for gDNA enumeration, and air-, or freeze-dried under anoxic conditions for mineralogical characterization.

Bentonite resin embedding for coupon-bentonite interface analysis. C-steel coupons, contained within bentonite sections measuring 1.5 to 2.0 cm in thickness, were sealed inside Mylar® bags within a glovebox (100% N_2) to ensure anoxic conditions. The samples were then frozen at -20°C and freeze-dried overnight. Dried samples were immediately transported to an anoxic glovebox. All next steps were then performed in the glovebox. First, EPO-TEK® 301-2, a two-component epoxy resin with low viscosity equilibrated with the glovebox atmosphere, was prepared according to the manufacturer's instructions for embedding the coupons. Silicone molds were coated with Vaseline® to enable the later removal of the resin. Next, a thin layer (approximately 3-4 mm) of resin was poured into the bottom of each mold and allowed to harden overnight. The next day, the freeze-dried samples were placed on top of this layer and covered with another portion of freshly prepared resin, with an excess applied to create a top layer of approximately 3-4 mm. The molds containing resin-embedded samples were then placed inside a desiccator connected to a pump. At least five vacuum-refill (N_2) cycles were conducted to remove all gas bubbles from bentonite, ensuring the resin penetrated the available pore spaces. Following this process, the samples were left to harden in the glovebox at least 48 hours. Finally, the resin-embedded bentonite samples containing the coupons were removed from the molds. They were then dry-cut using a saw under oxic conditions. After cutting, the samples were stored anoxically to limit O_2 exposure.

DNA quantification using a fluorometer. Extracted DNA was quantified using an Invitrogen Qubit 2.0 fluorometer with 2 µL of extracted DNA and 198 µL of a working solution prepared by mixing Qubit™ buffer solution and fluorescent Qubit™ reagent following the standard protocol provided by the manufacturer.

16S rRNA gene copy quantification using qPCR. Quantitative PCR (qPCR) of bacterial 16S gene copy numbers was performed using the MYRA robotic system and a MIC qPCR Cycler (both BioMolecular Systems, Australia) using10 µL reactions in triplicates. For quantification of gene abundance, the following volumes of substrates were used: 2.5 µL template DNA, 2.1 µL water, 0.2 µL of each primer (100 mM stock) and 5 µL of 2× SensiFAST SYBR ® No-ROX Kit (Meridian Bioscience, UK). Samples were cycled (40 cycles) at 95 °C for 5 s, followed by an extension at 62 °C for 10 s and the acquisition at 72 °C for 5 s. The final melting step was carried out from 72 °C to 95 °C, at a rate of 0.1 °C/s. Analysis of the results was performed using the built-in analytical software (micPCR, BioMolecular Systems, ver. 2.12.6). Average efficiency (0.943 – 1.003) and r^2 values (> 0.99) were determined from seven points of the serial dilutions (10^7 –10^1 copies) for bacterial 16S rRNA gene. Based on calibration curves obtained using *E. coli* DNA, C_q values were used to calculate the gene copy numbers which were normalized against the mass (ng) of the extracted DNA. The primer pair used for the reaction to quantify bacterial 16S rRNA gene consisted of 338f: 5'-ACT CCT ACG GGA GGC AGC AG-3' and 534r: 5'-ATT ACC GCG GCT GCT GGC A-3'.

HCl and HF Fe extraction. The freeze-dried outer layers of the cores, together with the reference materials (Wyoming bentonite (MX80), and gamma-irradiated Wyoming bentonite (MX80-S)) were subjected to HCl extractions to quantify and distinguish between different Fe pools. 0.5 M HCl is typically used to solubilize adsorbed and solid phase Fe(II) species, including siderite and green rust, and the reactive fraction of Fe(III) minerals [2], while 6 M HCl is used to extract more crystalline iron phases such as poorly reactive sheet silicate Fe or FeS species [3,4] Before extractions, all glassware was kept in a 10% HCl bath overnight and rinsed 3x with distilled water and 3x with MilliQ water. In an anoxic glovebox (100% N_2), 0.5 g of dry clay was weighed into a 50 mL serum bottle with 10 mL extractant and N_2 headspace. Throughout the extraction, all samples were kept in the dark for 24 h under anoxic conditions. Samples extracted with 0.5 M HCl were continuously shaken on a horizontal shaker at 100 rpm at room temperature, while samples mixed with 6 M HCl were kept anoxically in a 70 °C water bath. Following the extraction, 1 mL of sample was collected inside the glovebox at room temperature for 5 min at 13'500×g centrifugation, and 20 µL of supernatant was collected for 50x dilution in anoxic 1 M HCl. Fe(II) was quantified using the Ferrozine assay.[5] The

concentration of Fe(III) was calculated by subtracting Fe(II) from total Fe. Total Fe was obtained by HF digestions and quantified using the 1,10-phenanthroline assay as described earlier[6]. All experiments were performed in triplicate.

Preparation and handling of dithionite-reduced smectite suspensions. A 30 mM solution of Na_2S and a 30 mM solution of $Na_2S_2O_2$ were used to prepare the reference material of chemically reduced MX80 ($MX80_{red}$) following the method adapted from [7,8]. Briefly, in an anoxic glovebox (100% N_2), 1 g of powdered bentonite was suspended in 5 mL of citrate-bicarbonate buffer, and 30 mM of either Na_2S or 30 $Na_2S_2O_2$ was added. The vials were crimped to maintain anoxic conditions and allowed to react for 30 minutes in a 70°C water bath. Afterwards, the vials were brought back to the glovebox, and the supernatant, along with the particles, were transferred to several centrifugation tubes and centrifuged to remove the supernatant. The resulting reduced clay was washed with anoxic MQ in several (minimum 3) washing-centrifugation steps to remove residues of the salts from the supernatant and allowed to air-dry inside the glovebox. After drying, the powder was transferred to a vial and stored anoxically.

^{57}Fe Mössbauer Spectroscopy. Dried mineral powders were loaded into Plexiglas holders (area 1 cm^2), forming a thin disc. Holders were inserted into a closed-cycle exchange gas cryostat (Janis Cryogenics; now Lake Shore Cryotronics, Inc.) under a backflow of Helium to minimize exposure to air. Spectra were collected at 77K and 5K using a constant acceleration drive system (WissEL) in transmission mode with a ^{57}Co/Rh source. All spectra were calibrated against a 7 µm µm-thick α-^{57}Fe foil that was measured at room temperature. Analysis was carried out using Recoil fitting software (University of Ottawa) and the Voigt Based Fitting (VBF) routine.[9] The half width at half maximum (HWHM) was constrained to 0.124 mm/s during fitting.

Preparation of oriented samples for XRD analysis. Non-oriented samples were directly analyzed, while oriented mounts were prepared by deflocculation and decalcification of the bentonite sample, followed by slow sedimentation on a glass slide, allowing for the orientation of the clay crystals. Three oriented mounts were prepared for each sample: (1) air-dried, (2) glycolated, prepared by overnight incubation in a desiccator with an ethylene glycol-saturated atmosphere at 50°C, and (3) heated; prepared by heating the air-dried oriented sample up to 550°C for 1.5h.

Bentonite water content and dry density *calculations.* Water content was calculated following the equation:

$$\%\ water\ content = m_{water} \times 100/m_{sample\ dry}$$

where:

m_{water} - mass of water present in bentonite after borehole incubation (g), calculated as:

$$m_{water} = m_{sample\ wet} - m_{sample\ dry}$$

where:

$m_{sample\ dry}$ – dry weight, mass of bentonite sample after oven-drying (g)

$m_{sample\ wet}$ – wet weight, mass of bentonite sample after borehole incubation, before oven-drying (g)

Dry density (in g/cm^3) was calculated following the equation:

$$Density_{dry} = (m_{sample\ wet} - m_{water})/V_{mini\text{-}module}$$

where:

$V_{mini\text{-}module}$ – volume of the porous filter lining the min-module, calculated based on an average from three independent measurements of the inner diameter and height (in cm^3)

Corrosion analysis. The interface of metal coupons and bentonite, along with the thickness and specific features of the corrosion product layer (CPL), was investigated using a Laser

Confocal Optical Microscope Keyence (VK-X200) in depth composition mode, equipped with a Nikon objective (20x0.46, OFN25, WD-3.1 mm). To calculate the CPL thickness, a minimum of 3 images at 20x and at least of 30 measurements of CPL thicknesses were taken and analyzed with ImageJ software.[10] The Renishaw inViva Confocal Raman microscope with 532 nm laser and a 50X long-distance lens at optimum laser power in the range of 20 mW, was used to analyze the composition of the CPL.

Mass loss and corrosion rates. The C-steel coupon mass was measured before the experiment and after removing corrosion products accumulated during incubation in the borehole. The products were chemically removed following the standard procedures [11]. The dismantled coupons were washed with acetone in the ultrasonication for 2 minutes as a pre-step to remove loosely bound attached material/products. Thereafter, coupons were ultrasonically stirred in the solution containing 12.08 M HCl, 0.069 M Sb_2O_3 and 0.26 M $SnCl_2$ at room temperature for 2 minutes. The procedure was repeated until the weight of the coupons did not change between the cleaning steps. The corrosion rate was calculated as described below.

Corrosion rate calculations. The initial total surface area of the C-steel coupons delivered by Jacobs Engineering (Harwell Campus, UK; formerly Wood, AMEC Foster Wheeler) was recorded before the assembly of the modules. After the retrieval of the samples from the borehole, the mass loss was determined by subtracting the coupons' weight after removing corrosion products following the standard procedure (Methods), from the initial weight. The average corrosion rate was obtained as follows:

$$Corrosion\,rate = \frac{K \times W}{A \times T \times D}$$

where:

CR – corrosion rate (mm/year)
K – corrosion constant = $8.76{\times}10^4$ (correction factor to obtain CR in mm/year)
T – exposure time (h) = 13,140 h
A – coupon areas (cm^2) = 3.39 cm^2
W – mass loss (g)
D – C-steel density (g/ cm^3) = 7.86 g/ cm^3

2. Supplementary discussion

CPL and altered zone thicknesses. The inverse relationship between CPL and altered zone thicknesses is a consequence of the reaction of Fe^{2+}, released from C-steel, and its interaction with O_2 at the coupon–bentonite interface during the oxic abiotic corrosion formation stage (formation of the CPL precursor). In the 0% O_2 samples, where only trace O_2 was initially present, only a small portion of Fe^{2+} was oxidized at the interface. This led to a relatively thin CPL precursor, consisting mainly of Fe(III) (oxy)hydroxides, which were later transformed by Fe^{2+} into the mixed Fe^{2+}–Fe^{3+} phases observed in the CPL. The remaining Fe^{2+} that was neither oxidized nor participated in this transformation diffused further into the bentonite, producing a thicker altered zone. Conversely, in the 21% and 100% O_2 samples, a much larger pool of Fe^{2+} was immediately oxidized at the interface, forming a thicker CPL precursor. Because this thicker precursor required more Fe^{2+} for subsequent transformation into the observed mixed phases, less Fe^{2+} was available to migrate outward and alter the bentonite matrix. This led to the formation of a thinner altered zone.

3. Supplementary figures

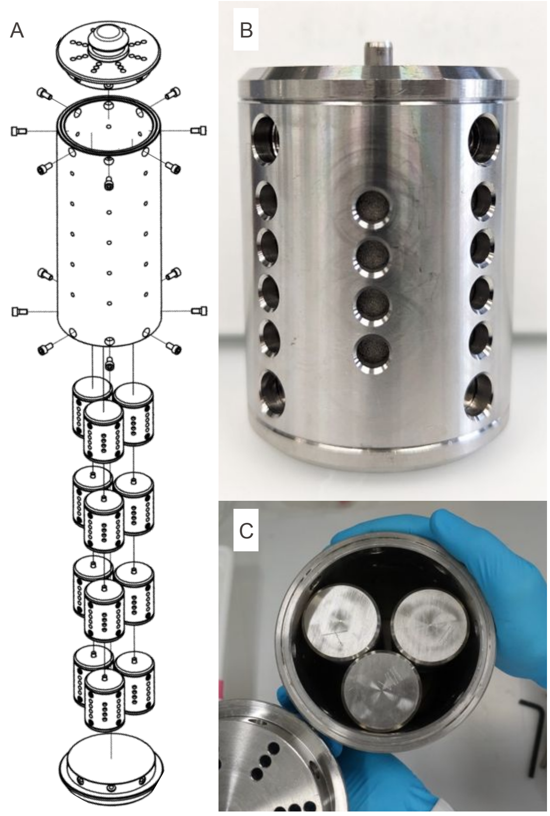

Figure S1. Experimental set-up**. A:** Technical drawing of a perforated stainless steel module housing 12 individual mini-modules. **B:** Photograph showcasing a single mini-module**. C:** Top view illustrating the arrangement of three mini-modules within the larger module.

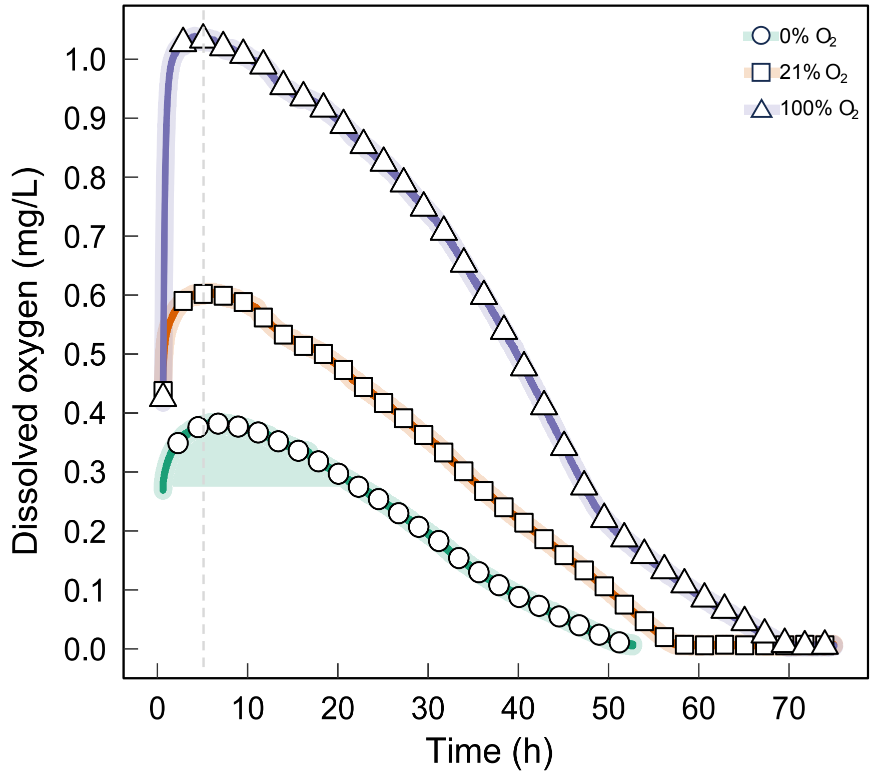

Figure S2. Oxygen dissolution in artificial pore water (APW) upon contact of bentonite equilibrated with 0% O_2 (green), 21% O_2 (orange) and 100% O_2 (purple) - containing atmosphere before *in-situ* incubation in the borehole (reference materials). The dashed vertical line represents the time needed for the maximum desorption of oxygen.

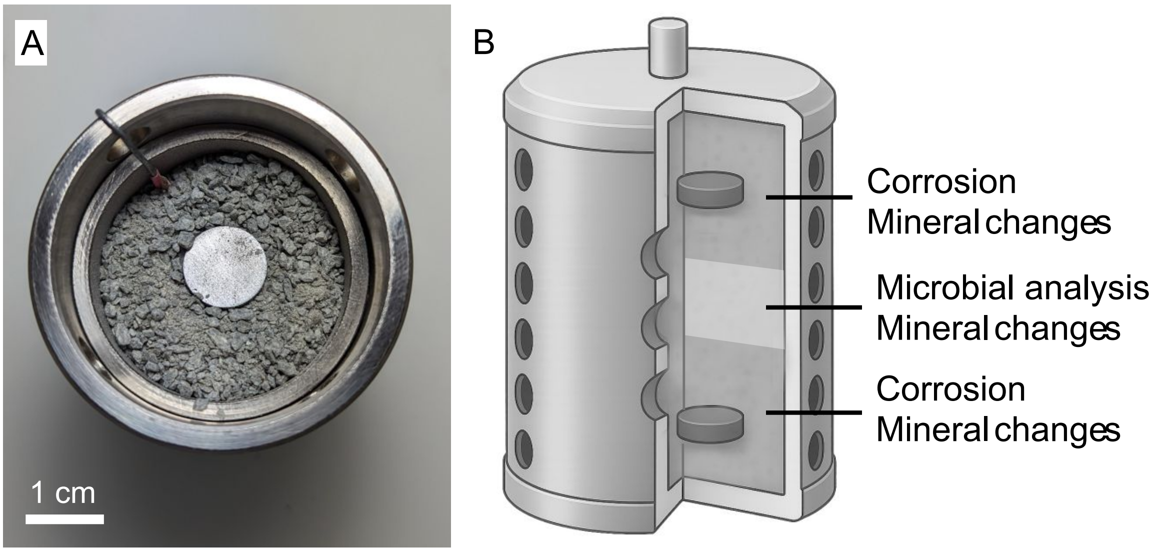

Figure S3. Assembly of the mini-module. **A:** Top view illustrating the mini-module filled with bentonite particles and the coupon placed in the middle. **B:** Mini-module diagram showing the placement of metal coupons and sectioning pattern: the coupon-containing layers (top and bottom) were used for studying the coupon-bentonite interface and corrosion, while the middle section was designated for microbial analysis (quantification and taxonomy identification) and mineral changes.

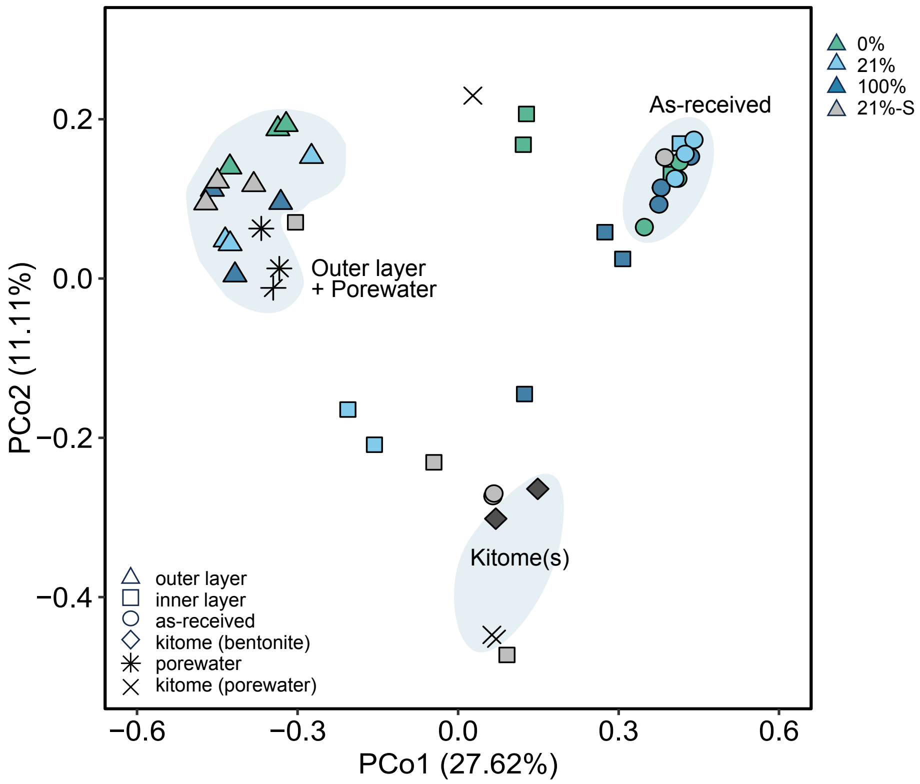

Figure S4. Principal Coordinate Analysis (PCoA) of 16S rRNA gene amplicon data for bentonite samples, porewater, as-received materials, and extraction kit controls (kitome). The inner and outer layer of the bentonite core is distinguished using different markers (see the legend). Different colors correspond to different concentrations of O_2 in the atmosphere used to equilibrate the samples.

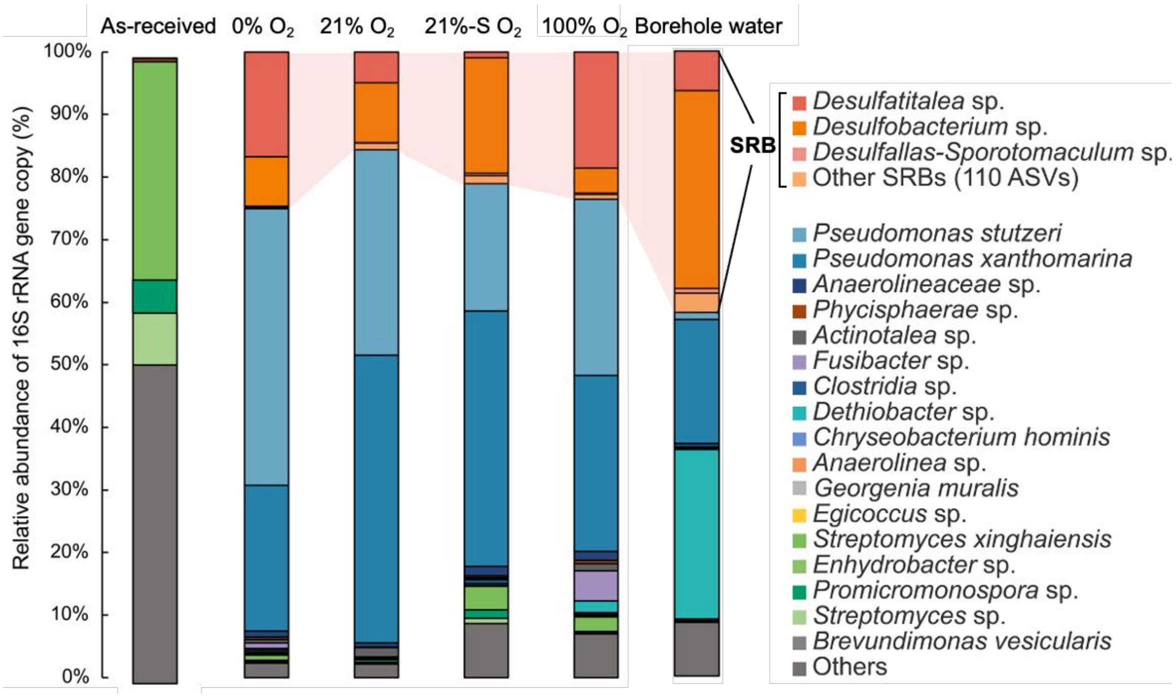

Figure S5. Microbial community composition based on full-length 16S rRNA gene amplicon sequencing representing borehole water, the outer parts of the bentonite core for each of the oxygen treatments, and as-received bentonite MX80. Species identified as sulfate-reducing bacteria (SRB) are shown in red-orange colors. The category 'Others' includes all amplicon sequence variants (ASVs) with an abundance of less than 2%.

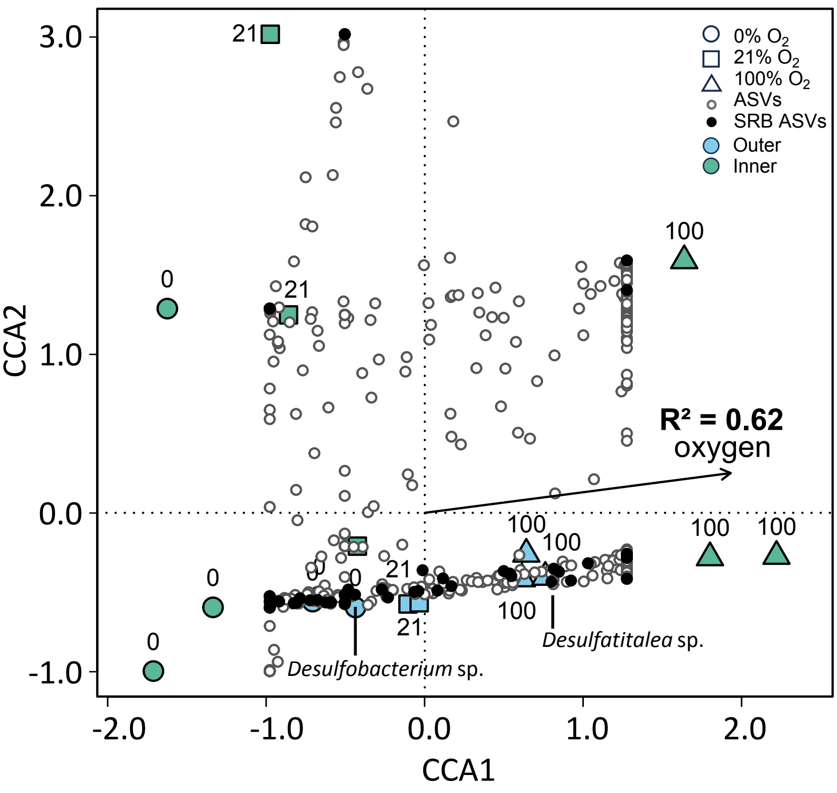

Figure S6. Canonical Correspondence Analysis (CCA) of microbial communities in bentonite, showing variation in relation to O_2 concentration to which bentonite was equilibrated before the deployment. Combined analysis of samples from both inner (green) and outer (blue) layers of the core. Shapes represent oxygen treatments: circles (0% O_2), squares (21% O_2), and triangles (100% O_2). Each small circle represents an ASV; sulfate-reducing bacteria (SRB)-related ASVS are shown in black, with the two most abundant SRBs labelled. The arrow indicates the direction and strength of the correlation between O_2 concentration and community composition. Oxygen has a stronger impact on the communities in the outer layer of the cores, which is indicated by the distribution of points representing ASVS and the difference in R^2 value when both outer and inner layer communities are included (R^2 = 0.89 vs. R^2 =, see Fig. 1).

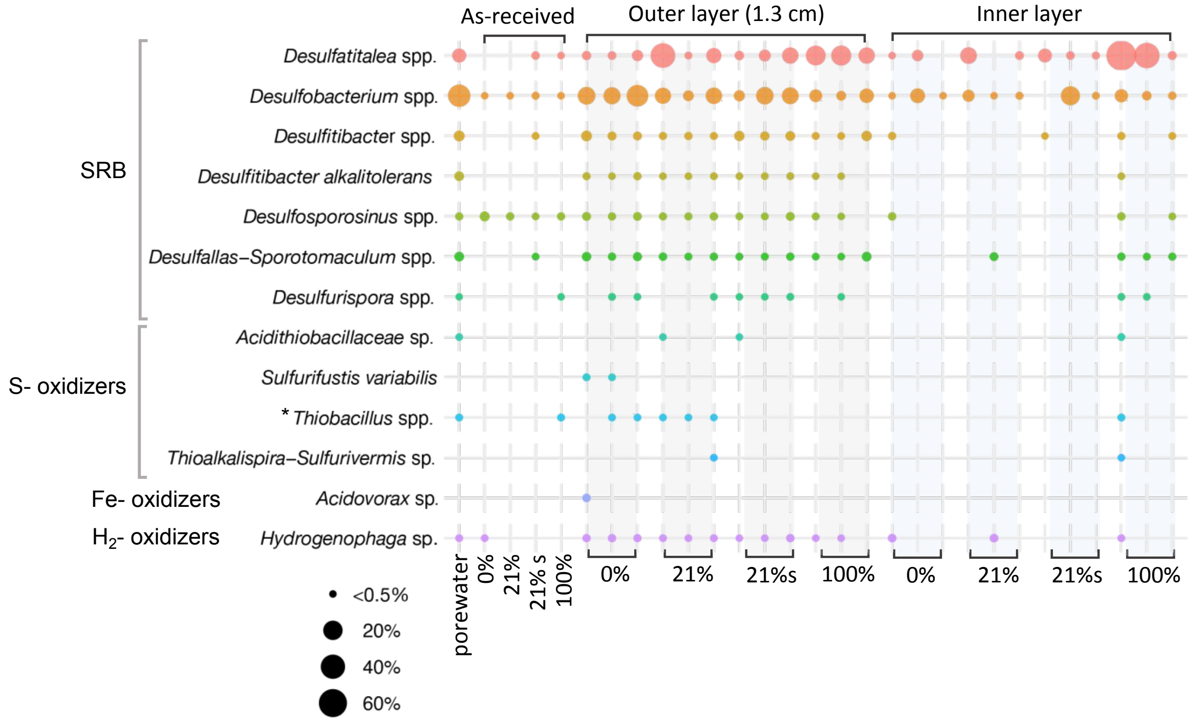

Figure S7. Bubble plot showing the relative abundance of ASV related to bacteria putatively metabolizing S-, Fe-species and H_2, identified in the as-received MX80 equilibrated to atmospheres varying in O_2 content (as-received bentonite), outer and inner bentonite layers. Different sizes of the bubbles correspond to varying abundance. *Thiobacillus* spp. are marked with (*), as they not only participate in S-oxidation but are also putative Fe(III)-reducers.

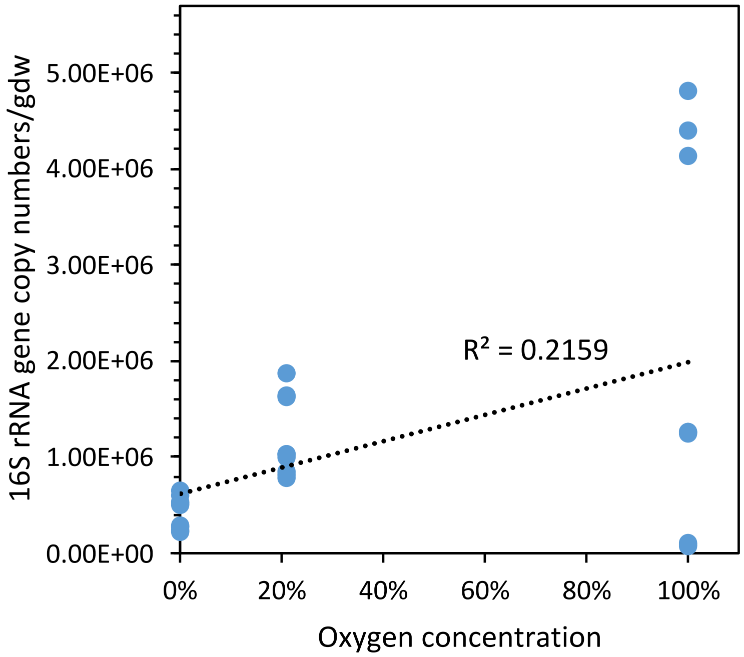

Figure S8. The distribution of 16S rRNA gene copy numbers normalized to grams of dry bentonite in relation to the oxygen concentration in the atmosphere used to equilibrate the bentonite prior to its deployment into the borehole. The dashed line indicates a positive linear correlation, with an R^2 value of 0.2159.

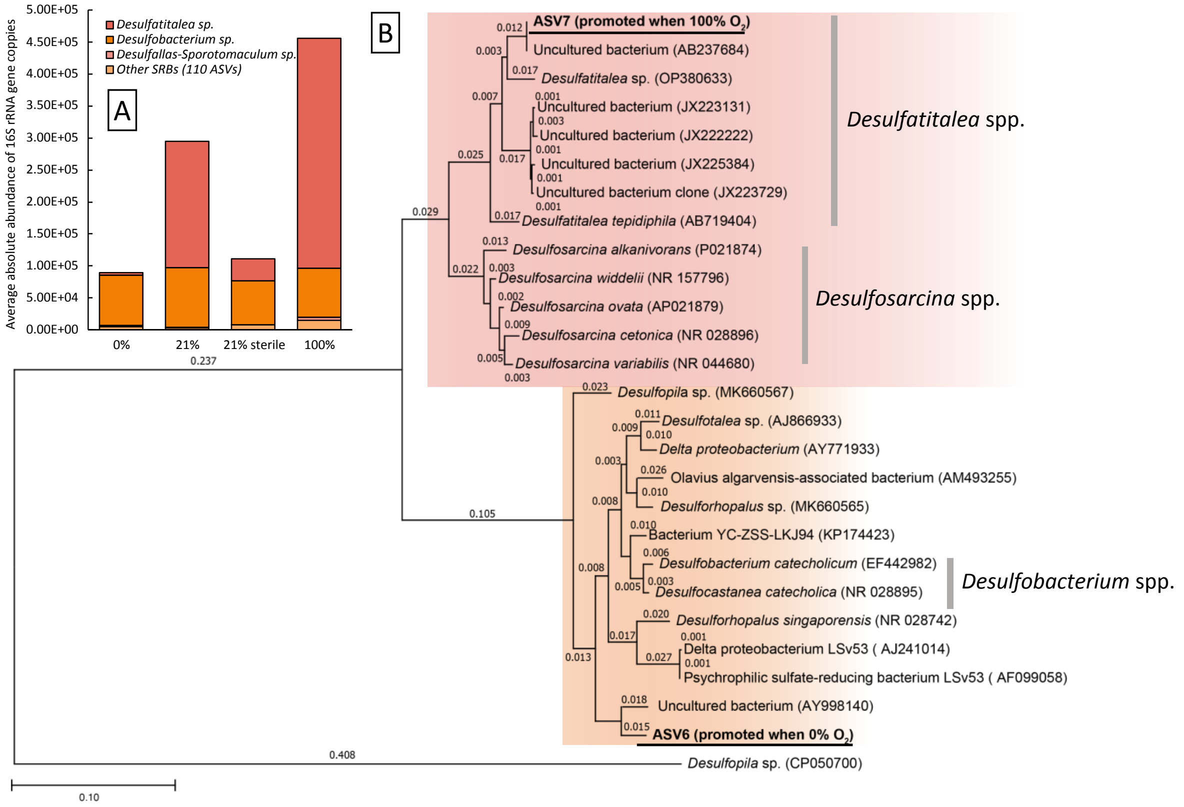

Figure S9. The absolute abundance **(A)** and taxonomy **(B)** of the two most abundant SRB: *Desulfurobacterium* sp (orange) and *Desulfatitalea* sp. (red) after 1.5 years of *in-situ* incubation in the borehole. The absolute abundance of 16S rRNA gene copies is represented by bars showing the average (3 samples for each treatment) SRB composition in the outer layer of the bentonite cores equilibrated with varying O_2 concentrations and the gamma-sterilized bentonite. The tree is constructed using maximum likelihood method. *Desulfopia* sp (CP050700), a common sulfate-reducing bacteria (SRB), was used as the outgroup.

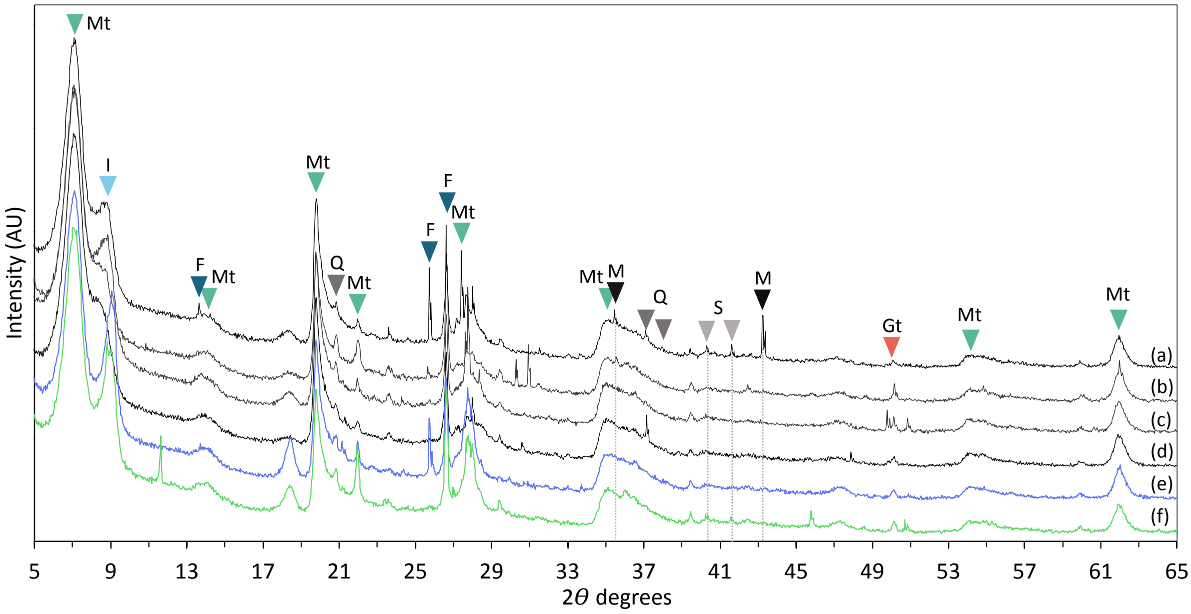

Figure S10. Powder X-ray diffraction patterns on the randomly oriented mounts. The patterns represent the outer layers of the bentonite cores: 0% O_2 (a), 21-S% O_2 (b), 21% O_2 (c), 100% O_2 (d), and the reference materials: as-received MX80 (e; blue line) and gamma-sterilized MX80 (f; green line). Triangles above the patterns represent characteristic peak positions of the minerals identified in the samples: montmorillonite (Mt), illite (I), feldspar (F), quartz (Q), and goethite (Gt) together with new mineral phases identified as magnetite (M) and siderite (S).

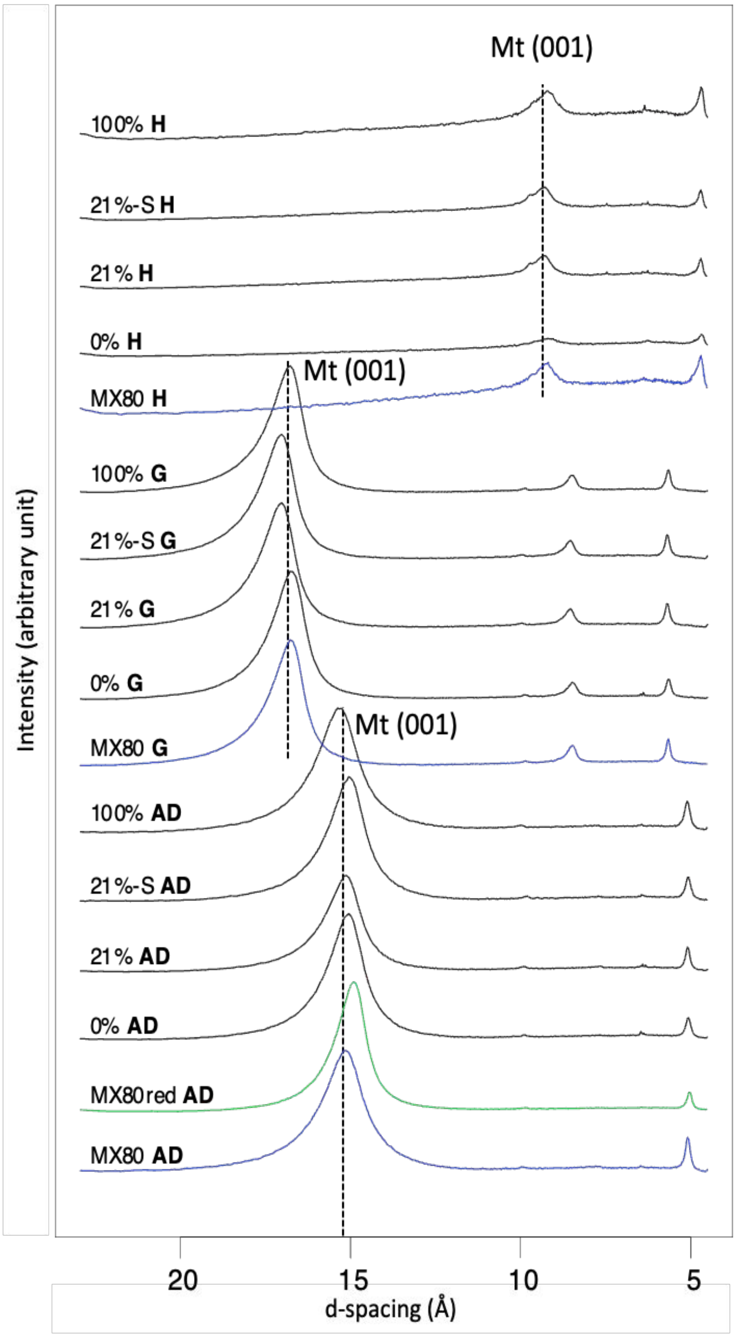

Figure S11. XRD patterns of oriented clay mounts following a step-procedure to identify clay minerals: air-dried (**AD**), expanded by ethylene glycol (glycolated; **G**), and heated (**H**) at 550°C. The dashed vertical line represents the position of the reflection from the basal 001 montmorillonite (Mt) plane. The samples also contain illite. The blue line represents untreated (as-received) MX80 bentonite. The green line represents MX80 bentonite chemically reduced with 30 mM Na_2S. No new clay phases were identified in the samples after the *in-situ* incubation.

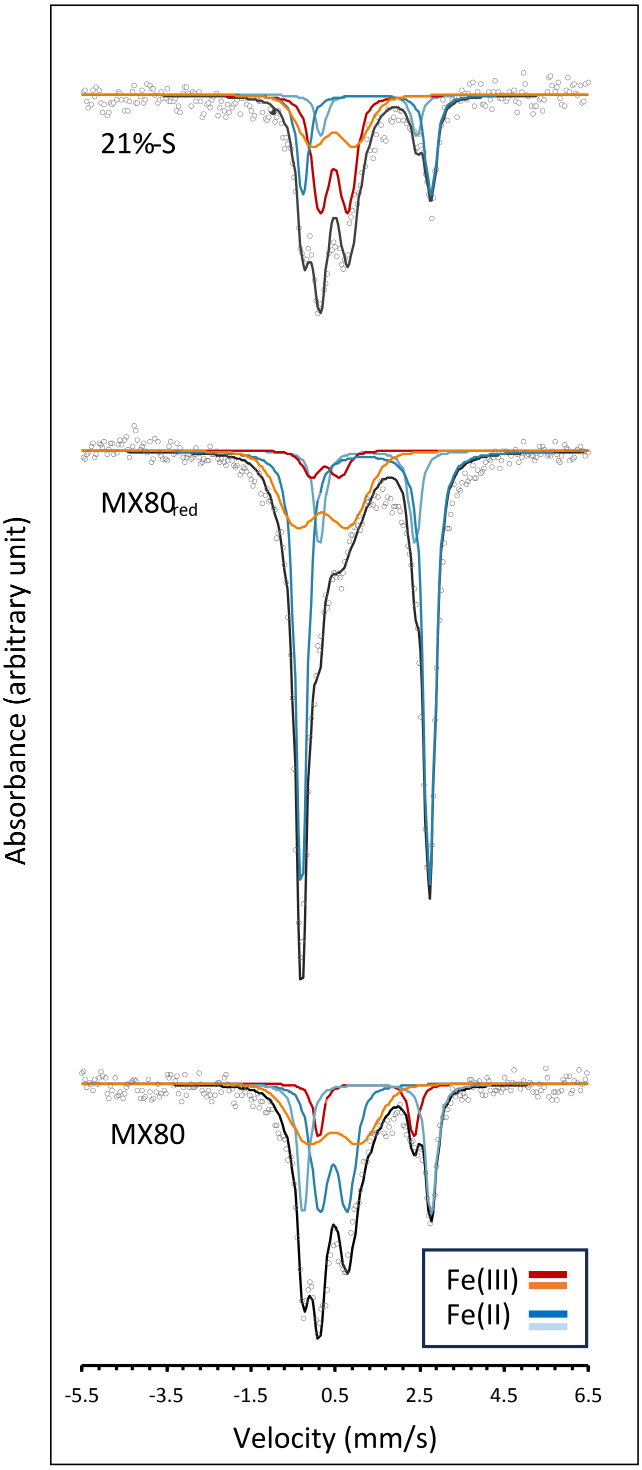

Figure S12. Mössbauer spectra (collected at 77 K) of two reference materials: as-received MX80 (MX80) and chemically reduced MX80 ($MX80_{red}$), and a gamma-sterilized bentonite sample equilibrated with 21% O_2 incubated *in-situ* incubation (21%-S). Empty circles represent raw data, while the black line shows the fitted spectrum. Blue lines represent fitted Fe(**II**) doublets, while orange-red lines correspond to fitted Fe(**III**) doublets.

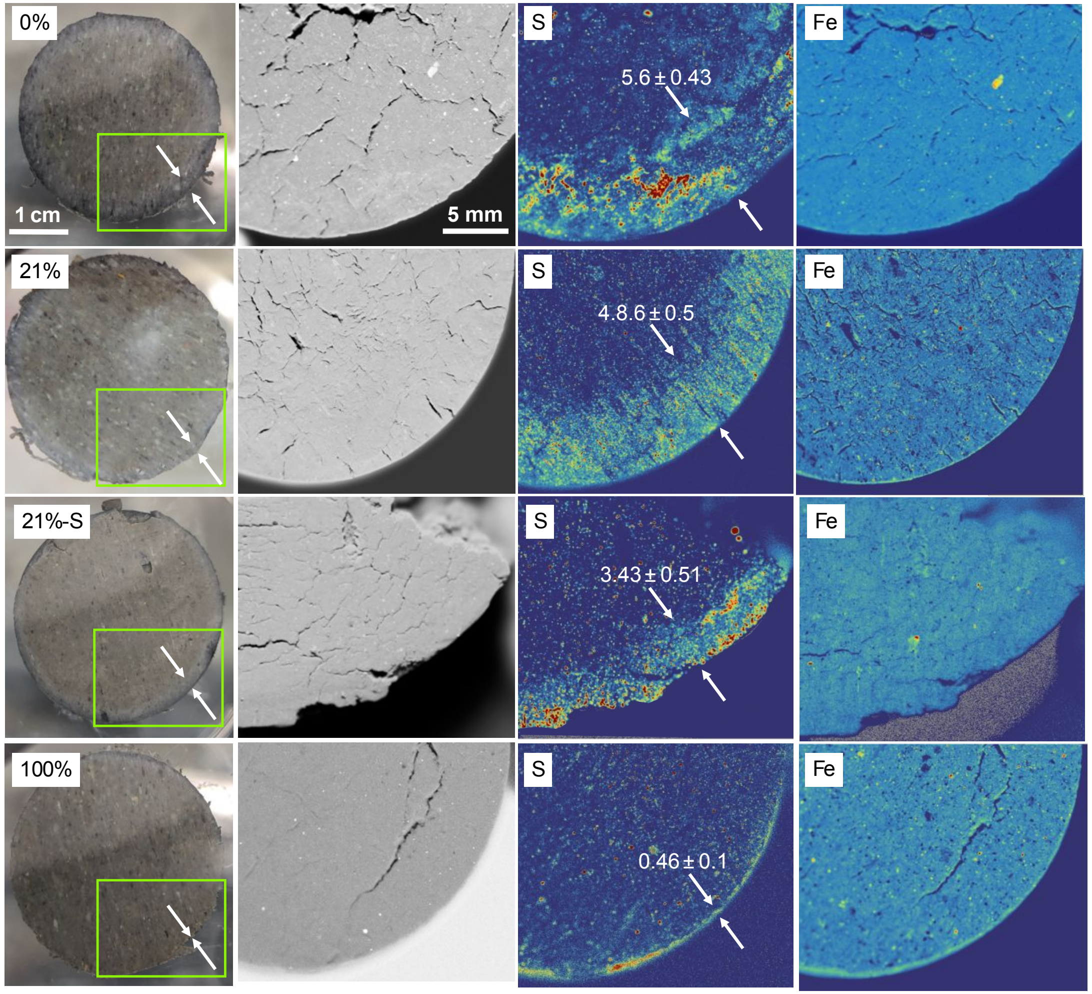

Figure S13. Macroscopic and microscopic pictures and XRF elemental distribution maps showing S and Fe distribution in the outer layer of the bentonite cores. Horizontal panels represent (from the top): 0% O_2, 21% O_2 gamma-irradiated (sterile), 21% O_2 and 100% O_2 bentonite treatments. Accumulation of S represented as the distribution of bright blue spots in the last column, differs between the treatments reflecting the sulfate reduction front within the bentonite and the contact with sulfate-containing

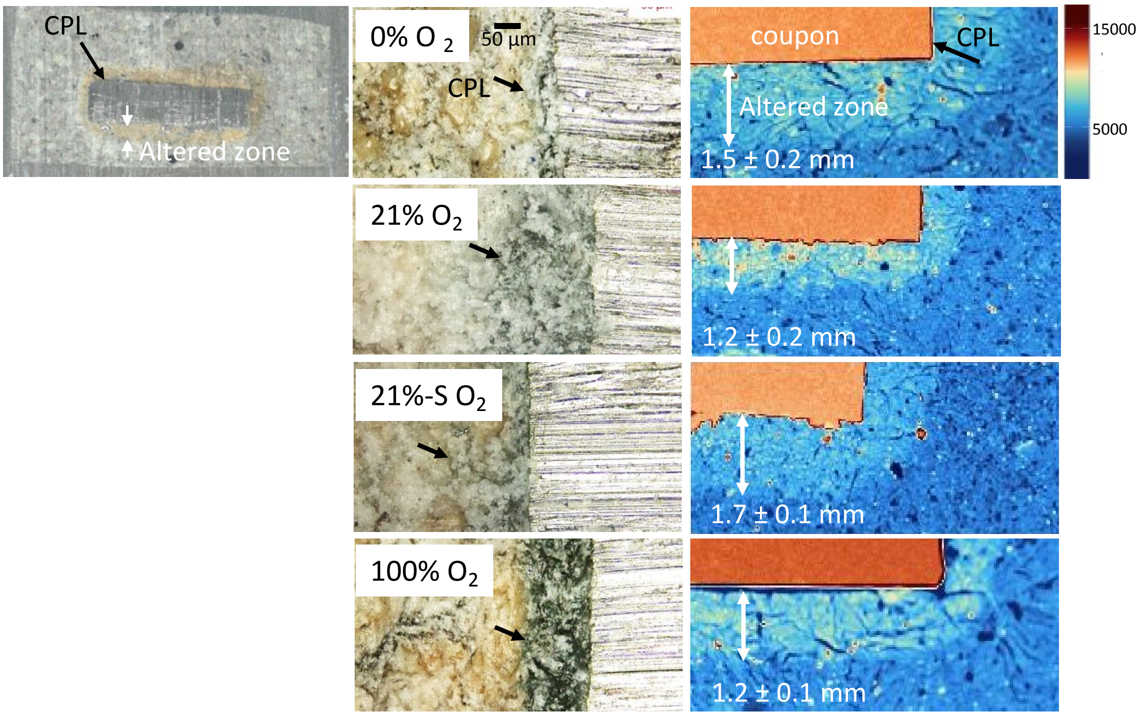

Figure S14. Confocal microscopy images and XRF maps illustrate the cross-sectional analysis of the resin-embedded coupon-bentonite interface. The black arrows indicate the Corrosion Product Layer (CPL), while the white arrows indicate the altered zone, with numerical values representing the bentonite altered zone thickness (n>15) in mm. Elemental distribution maps show iron (Fe) localization around the carbon steel coupon, with horizontal panels depicting different treatment conditions: 0% O_2, 21% O_2 and 21% O_2 gamma-irradiated (sterile), and 100% O_2.

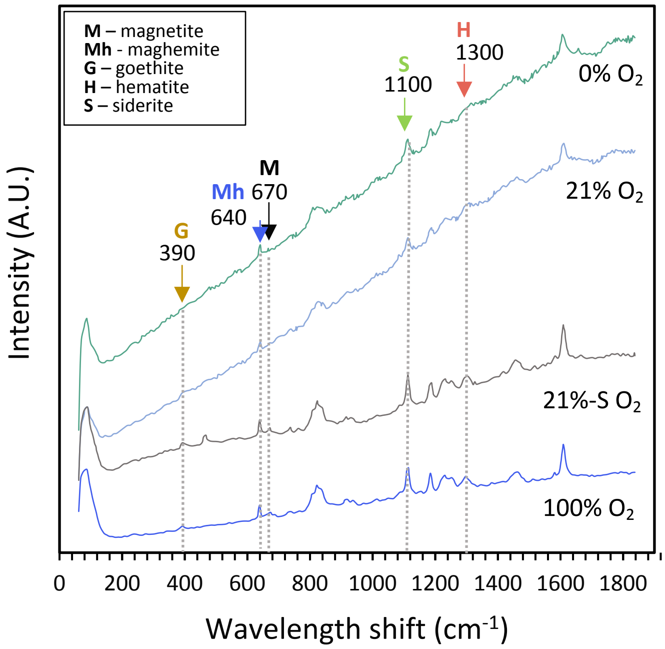

Figure S15. µRaman spectra of CPL showing corrosion product. Letters, arrows and dotted lines indicate the characteristic peak positions for magnetite (**M**), maghemite (**Mh**), goethite (**G**), hematite (**H**) and siderite (**S**) identified in the sample.

STEP 1: Saturation and bentonite swelling

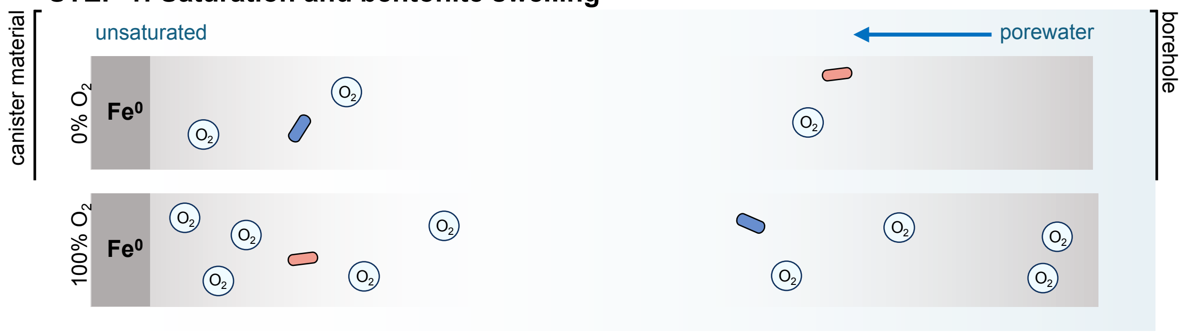

- Saturation starts. Advective flow of porewater.
- Indigenous bentonite bacteria are inactive.

STEP 2: Oxic corrosion and bacterial colonization

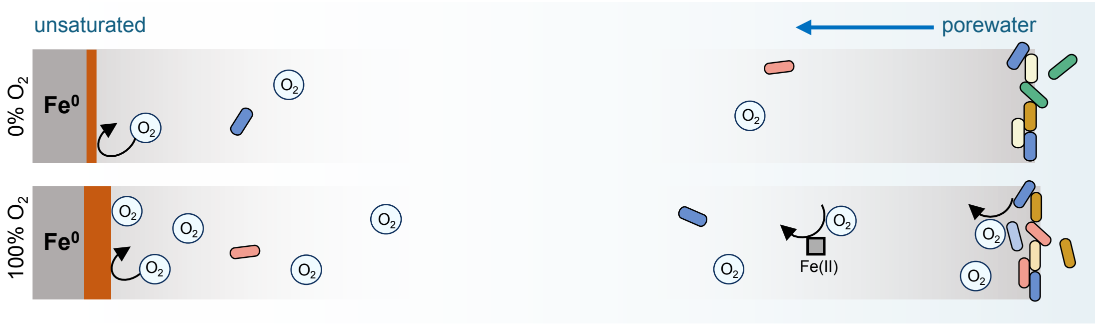

- Formation of primary Fe(III) (oxy)hydroxides (**oxic corrosion**) happens due to the abiotic reaction with O_2 in the presence of water present in the as-received bentonite.
- The smaller thickness at **0% O_2** results from less O_2 (trace amounts adsorbed to the bentonite).
- Porewater bacterial cells colonize bentonite until it fully swells (and pore space is insufficient)
- O_2 dissolves immediately in contact with porewater, leading to the formation of unique microbial assemblages. Biotic O_2 consumption.
- Abiotic O_2 depletion due to the oxidation of Fe(II)-phases.

STEP 3: Anoxic corrosion and sulfate reduction

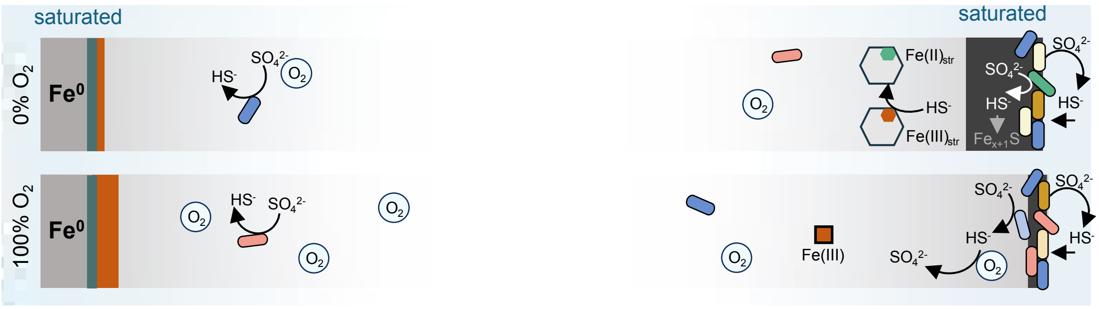

- Formation of Fe^{2+} through **anoxic corrosion** by the reduction of water and the production of H_2.

$$Fe^0 \rightarrow Fe^{2+} + 2e^-$$

$$H_2O + e^- \rightarrow OH^- + \tfrac{1}{2}H_2\uparrow$$

- Locally, indigenous bentonite SRB reduce SO_4^{2-}.
- Activity of SRB. Production of HS^-. Precipitation of mackinawite or pyrite ($Fe_{1+x}S$).
- **100% O_2**: thinner $Fe_{1+x}S$ layer results from the re-oxidation of HS^- by residual O_2. Thus, less Fe(II) available to form $Fe_{1+x}S$.
- **0% O_2:** Abiotic reduction of Fe(III), incl. $Fe(III)_{str}$ in montmorillonite by HS^-.

STEP 4: Corrosion product layer (CPL) formation

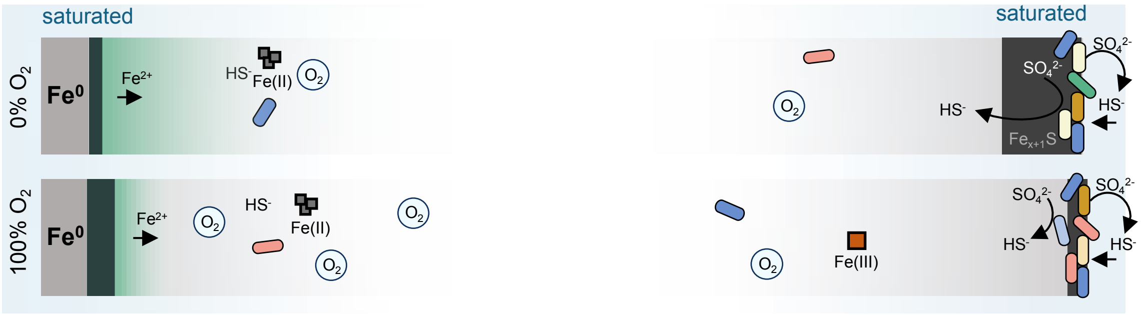

- Diffusion of Fe^{2+} into the clay through the primary Fe(III) (oxy)hydroxides layer.
- **DPL formation** via the transformation of Fe(III) (oxy)hydroxides into magnetite (e.g., Schikkor reaction).
- **100% O_2:** Fe^{2+} travels a short distance because: (1) more is used to transform the thicker primary (oxy)hydroxides layer, and (2) of the slower diffusion through the thicker DPL.
- Continuous production of HS^- at the boundary with the host rock (porewater) if the porosity is sufficient to allow for bacterial activity.
- Diffusion of HS^- inside bentonite.

STEP 5: Altered betnonite zone formation

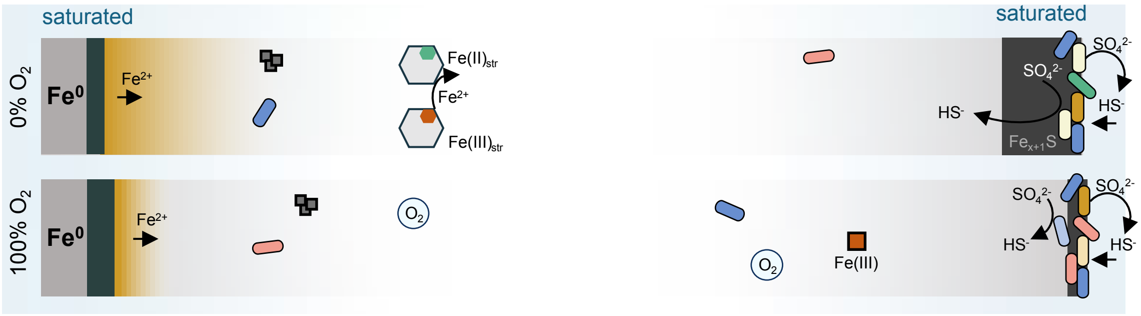

- **TM formation:** oxidation of Fe^{2+} in contact with residual O_2. Formation of Fe(III) (oxy)hydroxides.
- **100% O_2:** The thinner TM is due to the shorter distance Fe^{2+} could travel (see the previous step).
- Indigenous bentonite bacteria activity is limited, or they become inactive.
- If O_2 absent: continuous diffusion of Fe^{2+} and Fe^{2+}-induced changes of Fe(III)-bearing phases.
- Continuous production of HS^- at the boundary with the host rock (porewater) if the porosity is sufficient to allow for bacterial activity.
- Diffusion of HS^- inside bentonite.

Figure S16. Conceptual model depicting the formation of corrosion products of carbon steel and the microbial activity within bentonite. The upper panel illustrates samples equilibrated at 0% O_2, while the lower panel corresponds to samples equilibrated at 100% O_2. The schematics show two regions: the near-field, next to the canister material (left), and the far-field, represented by the bentonite in direct contact with porewater and the Opalinus Clay host rock.

4. Supplementary tables

Table S1. Fe chemical extraction data from bentonite samples using 0.5 M HCl, 6 M HCl and hydrofluoric acid (HF).

Solvent	Fe pool	0% O_2 mg/gdw[b]	21% O_2 mg/gdw	21% O_2-S mg/gdw	100% O_2 mg/gdw	MX80 mg/gdw	MX80-S mg/gdw	Na_2S-reduced[c] mg/gdw	$Na_2S_2O_4$ reduced[d] mg/gdw
0.5 M HCl	Fe(II)	1.88	2.16	1.95	2.42	1.81	1.48	15.63	16.10
	Fe(III)	0.00	0.00	0.00	0.00	0.00	0.00	0.00	0.00
	Total $Fe_{0.5M\ HCl}$[a]	1.88	2.16	1.95	2.42	1.81	1.48	15.63	16.10
6 M HCl	Fe(II)	8.62	6.72	6.57	5.12	3.31	2.80	14.07	14.49
	Fe(III)	4.20	0.70	1.89	2.40	3.85	5.22	0.00	0.00
	Total $Fe_{6M\ HCl}$[a]	12.82	7.43	8.45	7.53	7.17	8.02	14.07	14.49
HF	Fe(II)	10.32	7.00	5.47	4.97	2.58	n.d.	n.d.	n.d.
	Fe(III)	13.55	14.56	18.67	16.84	22.92	n.d.	n.d.	n.d.
	Total Fe_{HF}[a]	23.87	21.56	24.14	21.81	25.50	n.d.	n.d.	n.d.
HF	Fe(II)/Fe(III)	0.76	0.48	0.29	0.30	0.11	n.d.	n.d.	n.d.

[a]calculated as the sum of Fe(II) and Fe(III)
[b]mg per g of dry bentonite weight
[c]Wyoming MX80 bentonite chemically reduced with 30 mM Na_2S, elsewhere referred to as $MX80_{red}$
[d]Wyoming MX80 bentonite chemically reduced with 30 mM $Na_2S_2O_4$

Table S2. Mössbauer spectroscopy hyperfine parameters collected, including center shift (CS), quadrupole splitting (QS), hyperfine field (H), sigma, the relative area and the goodness of the fit χ^2.

§	Phase	CS (mm/s)	QS/ ε (mm/s)	H (T)	Σ	Relative area (%)	χ^2
MX80 (77K)	Fe(II) doublet	1.23	2.26		0.09	9.8	*1.16*
	Fe(II) doublet	1.26	3.03		0.12	25.6	
	Fe(III) doublet	0.46	0.65		0.29	33.5	
	Fe(III) doublet	0.47	1.25		0.72	31.1	
$MX80_{red}$[a] (77K)	Fe(II) doublet	1.24	2.26		0.12	12.0	*1.32*
	Fe(II) doublet	1.25	3.03		0.13	57.4	
	Fe(III) doublet	0.39	0.66		0.29	4.7	
	Fe(III) doublet	0.24	1.25		0.72	26.0	
0% (77K)	Fe(II) doublet	1.26	3.04		0.11	46.0	*0.88*
	Fe(II) doublet	1.24	0.28		0.09	5.4	
	Fe(III) doublet	0.44	0.63		0.25	26.8	
	Fe(III) doublet	0.45	1.02		0.56	21.8	
21% (77K)	Fe(II) doublet	1.29	2.26		0.09	9.2	*1.01*
	Fe(II) doublet	1.25	3.03		0.12	35.1	
	Fe(III) doublet	0.47	0.65		0.29	29.5	
	Fe(III) doublet	0.46	1.02		0.56	26.2	
21%-S (77K)	Fe(II) doublet	1.29	2.26		0.09	9.6	*0.83*
	Fe(II) doublet	1.25	3.03		0.12	24.9	
	Fe(III) doublet	0.47	0.65		0.29	38.6	
	Fe(III) doublet	0.46	1.02		0.56	27.0	
100% (77K)	Fe(II) doublet	1.40	2.26		0.09	9.0	*0.97*
	Fe(II) doublet	1.24	3.06		0.12	22.4	
	Fe(III) doublet	0.48	0.61		0.28	32.1	
	Fe(III) doublet	0.46	1.02		0.56	36.5	

[a]Wyoming MX80 bentonite chemically reduced with 30 mM Na_2S

Table S3. Data comparison between the relative percentage of total Fe(II) and total Fe(III) in the samples obtained by two different methods: hydrofluoric (HF) acid digestion and Mössbauer spectroscopy. For the latter, the sum of Fe(II) and Fe(III) was obtained by the addition of the respective relative areas of Fe(II) and Fe(III) listed in detail in Table S2.

	HF acid digestion		Mössbauer spectroscopy	
**Sample**	**Fe(II) (%)**	**Fe(III) (%)**	**Fe(II) relative area (sum; %)**	**Fe(III) relative area (sum; %)**
MX80	10.20	89.80	30.6	69.4
$MX80_{red}$[a]	n.d.	n.d.	51.4	48.6
0%	43.10	56.90	44.3	55.7
21%	32.41	67.59	34.5	65.5
21%-S	22.82	77.18	31.4	68.6
100%	22.94	77.06	35.4	64.6

[a]Wyoming MX80 bentonite chemically reduced with 30 mM Na_2S

Table S4. Corrosion of C-steel coupons inside compacted bentonite equilibrated with 0%, 21% and 100% O_2-containing atmosphere and in an anoxic borehole for 1.5 years. Abiotic control (gamma-sterilized bentonite) was equilibrated with a 21% O_2-containing atmosphere. The results represent the thickness of the corrosion product layer (CPL) and altered zone, mass loss over the course of 1.5 years of *in-situ* incubation and mass-loss corrosion rates per year.

Sample	CPL thickness (µm)	Altered zone thickness (mm)	Mass loss (g)	Corrosion rate (µm/year)
0% O_2	38.1±19.2	1.5±0.2	0.010	2.50
21% O_2	121.5±79.7	1.2±0.1	0.009	2.25
21%-S O_2	72.0±19.3	1.7±0.1	0.011	2.75
100% O_2	96.5±33.9	1.2±0.2	0.017	4.25

Table S5. Pairwise Student's *t*-tests with Bonferroni correction showing differences in 16S rRNA gene copy numbers between O_2 treatments in outer and inner bentonite core layers. Raw *p*-values are reported; significance is indicated by asterisks ns = not significant, $p < 0.05$ (*), $p < 0.01$ (**), $p < 0.001$ (***).

Core layer	Pair	p-value	k (comparisons)	Bonferroni α	Adjusted p-value	Significant (Bonferroni)
outer	0% O_2 & 21% O_2-S	0.05	6	0.008333	0.002698	**
outer	0% O_2 & 21% O_2	0.05	6	0.008333	0.000118	***
outer	0% O_2 & 100% O_2	0.05	6	0.008333	0.035298	ns
outer	21% O_2-S & 21% O_2	0.05	6	0.008333	0.007744	*
outer	21% O_2-S & 100% O_2	0.05	6	0.008333	0.083292	ns
outer	21% O_2 & 100% O_2	0.05	6	0.008333	0.276575	ns
inner	0% O_2 & 21% O_2-S	0.05	6	0.008333	0.01052	ns
inner	0% O_2 & 21% O_2	0.05	6	0.008333	0.373851	ns
inner	0% O_2 & 100% O_2	0.05	6	0.008333	0.128686	ns
inner	21% O_2-S & 21% O_2	0.05	6	0.008333	0.034954	ns
inner	21% O_2-S & 100% O_2	0.05	6	0.008333	0.422373	ns
inner	21% O_2 & 100% O_2	0.05	6	0.008333	0.422373	ns
inner	0% O_2 & 0% O_2 as-received	0.05	4	0.0125	0.005567	*
inner	21% O_2 & 21% O_2 as-received	0.05	4	0.0125	0.587547	ns
inner	21% O_2-S & 21% O_2-S γ-sterilized	0.05	4	0.0125	2.56E-05	***
inner	100% O_2 & 100% O_2 as-received	0.05	4	0.0125	0.100127	ns

5. Supplementary references

(1) Pearson, F. J. *Opalinus Clay Experimental Water: A1Type, Version 980318*; Villigen PSI, Switzerland, 1998.

(2) Fredrickson, J. K.; Zachara, J. M.; Kennedy, D. W.; Dong, H.; Onstott, T. C.; Hinman, N. W.; Li, S. M. Biogenic Iron Mineralization Accompanying the Dissimilatory Reduction of Hydrous Ferric Oxide by a Groundwater Bacterium. *GeCoA* **1998**, *62* (19), 3239–3257. https://doi.org/10.1016/S0016-7037(98)00243-9.

(3) Heron, G.; Crouzet, C.; Bourg, A. C. M.; Christensen, T. H. Speciation of Fe(II) and Fe(III) in Contaminated Aquifer Sediments Using Chemical Extraction Techniques. *Environ Sci Technol* **1994**, *28* (9), 1698–1705. https://doi.org/10.1021/ES00058A023/.

(4) Poulton, S. W.; Canfield, D. E. Development of a Sequential Extraction Procedure for Iron: Implications for Iron Partitioning in Continentally Derived Particulates. *Chem Geol* **2005**, *214* (3–4), 209–221. https://doi.org/10.1016/J.CHEMGEO.2004.09.003.

(5) Stookey, L. L. Ferrozine-A New Spectrophotometric Reagent for Iron. *Anal Chem* **1970**, *42* (7), 779–781. https://doi.org/10.1021/AC60289A016.

(6) Amonetie, J. E.; Templeton, J. C. *Improvements to the Quantitative Assay of Nonrefractory Minerals for Fe(II) and Total Fe Using 1,10-Phenanthroline*; 1998; Vol. 46.

(7) Ilgen, A. G.; Kukkadapu, R. K.; Leung, K.; Washington, R. E. "Switching on" Iron in Clay Minerals. *Environ Sci Nano* **2019**, *6* (6), 1704–1715. https://doi.org/10.1039/C9EN00228F.

(8) Stucki, J. W. The Quantitative Assay of Minerals for Fe2+ and Fe3+ Using 1,10-Phenanthroline—II: A Photochemical Method. *Soil Sci. Soc. Amer. J.* **1981**, *45* (3), 638–641. https://doi.org/10.2136/sssaj1981.03615995004500030040x.

(9) Lagarec, K.; Rancourt, D. G. Extended Voigt-Based Analytic Lineshape Method for Determining N-Dimensional Correlated Hyperfine Parameter Distributions in Mössbauer Spectroscopy. *Nucl Instrum Methods Phys Res B* **1997**, *129* (2), 266–280. https://doi.org/10.1016/S0168-583X(97)00284-X.

(10) Rueden, C. T.; Schindelin, J.; Hiner, M. C.; DeZonia, B. E.; Walter, A. E.; Arena, E. T.; Eliceiri, K. W. ImageJ2: ImageJ for the next Generation of Scientific Image Data. *BMC Bioinformatics* **2017**, *18* (1). https://doi.org/10.1186/S12859-017-1934-Z.

(11) ASTM International. Standard Practice for Preparing, Cleaning, and Evaluating Corrosion Test Specimens. *Standard G1-03, ASTM Int., West Conshohocken, PA, USA.* **2017**.
